# Measuring malaria diagnosis and treatment coverage in population-based surveys: a recall validation study in Mali among caregivers of febrile children under 5 years

**DOI:** 10.1186/s12936-018-2636-3

**Published:** 2019-01-03

**Authors:** Ruth A. Ashton, Bakary Doumbia, Diadier Diallo, Thomas Druetz, Lia Florey, Cameron Taylor, Fred Arnold, Jules Mihigo, Diakalia Koné, Seydou Fomba, Erin Eckert, Thomas P. Eisele

**Affiliations:** 1 MEASURE Evaluation, Center for Applied Malaria Research and Evaluation, Tulane School of Public Health and Tropical Medicine, Tulane University, 1440 Canal Street, Suite 2300, New Orleans, LA USA; 2 Info-Stat, Bamako, Mali; 3 MEASURE Evaluation, ICF, Bamako, Mali; 4 Center for Applied Malaria Research and Evaluation, Tulane School of Public Health and Tropical Medicine, New Orleans, LA USA; 5 Department of Social and Preventive Medicine, School of Public Health, University of Montreal, Montreal, QC Canada; 6 President’s Malaria Initiative, United States Agency for International Development, Washington, DC USA; 7 The DHS Program, ICF, Rockville, MD USA; 8 President’s Malaria Initiative, United States Agency for International Development, Bamako, Mali; 9 Programme National de Lutte contre le Paludisme, Bamako, Mali

**Keywords:** Population survey, Malaria, Artemisinin-based combination therapy, Recall validation

## Abstract

**Background:**

Nationally-representative household surveys are the standard approach to monitor access to and treatment with artemisinin-based combination therapy (ACT) among children under 5 years (U5), however these indicators are dependent on caregivers’ recall of the treatment received.

**Methods:**

A prospective case–control study was performed in Mali to validate caregivers’ recall of treatment received by U5s when seeking care for fever from rural and urban public health facilities, community health workers and urban private facilities. Clinician-recorded consultation details were the gold standard. Consenting caregivers were followed-up for interview at home within 2 weeks using standard questions from Demographic and Health Surveys and Malaria Indicator Surveys.

**Results:**

Among 1602 caregivers, sensitivity of recalling that the child received a finger/heel prick was 91.5%, with specificity 85.7%. Caregivers’ recall of a positive malaria test result had sensitivity 96.2% with specificity 59.7%. Irrespective of diagnostic test result, the sensitivity and specificity of caregivers’ recalling a malaria diagnosis made by the health worker were 74.3% and 74.9%, respectively. Caregivers’ recall of ACT being given had sensitivity of 43.2% and specificity 90.2%, while recall that any anti-malarial was given had sensitivity 59.0% and specificity 82.7%. Correcting caregivers’ response of treatment received using a combination of a visual aid with photographs of common drugs for fever, prescription documents and retained packaging changed ACT recall sensitivity and specificity to 91.5% and 71.1%, respectively.

**Conclusions:**

These findings indicate that caregivers’ responses during household surveys are valid when assessing if a child received a finger/heel prick during a consultation in the previous 2 weeks, and if the malaria test result was positive. Recall of ACT treatment received by U5s was poor when based on interview response only, but was substantially improved when incorporating visual aids, prescriptions and drug packaging review.

**Electronic supplementary material:**

The online version of this article (10.1186/s12936-018-2636-3) contains supplementary material, which is available to authorized users.

## Background

To assess coverage of recommended first-line anti-malarial drugs, malaria control programs often draw on data from large-scale population-based household surveys, such as the Demographic and Health Survey (DHS) and Malaria Indicator Survey (MIS) [1–3]. The standard indicator used to assess if treatment guidelines are being adhered to is the proportion of children receiving ACT (or other recommended treatment) among children under 5 years (U5) with fever in the last 2 weeks who received any anti-malarial drugs [3]. This estimate of ACT coverage is used by National Malaria Control Programmes to understand whether children are receiving appropriate treatment for uncomplicated malaria when seeking care from public health facilities, community health workers and private health services. In addition, this indicator contributes to analysis estimating global estimates of diagnosis and treatment services, performed by the World Health Organization (WHO) and other international organizations [4, 5].

The proportion of U5s with fever in the last 2 weeks who had a finger or heel prick is commonly collected in population-based surveys to provide information about access to confirmatory diagnostics for malaria at the locations where caregivers seek care for the child. However, further questions such as whether caregivers were told the result of the test, if they remember the result of the test, and if the caregivers were told the final diagnosis made by the health worker are not routinely included in population surveys, but have the potential to further contribute to monitoring of malaria diagnosis and treatment practices if found to be valid.

The potential for bias in caregiver recall of diagnosis and treatment of febrile children was among multiple challenges identified in the use of population-based surveys to measure key maternal and child health indicators [6]. A validation study at public health facilities in Zambia found that caregiver recall of ACT received by a febrile child under 5 years of age had high sensitivity and specificity [7]. Zambia has successfully limited the use of monotherapies for malaria treatment [8], and has relatively low levels of treatment-seeking from the private sector [9], therefore, there is a need to replicate this study in settings where a wider range of drugs could potentially be prescribed for malaria, and among caregivers attending private sector facilities.

Mali was identified as an appropriate location to further explore the validity of caregiver recall of ACT received by children under five. Findings in recent household surveys in Mali indicate that caregivers reported non-ACT drugs such as amodiaquine, sulfadoxine-pyrimethamine (SP) or chloroquine were given to their sick children for uncomplicated malaria during visits to health facilities [10, 11]. Recent introduction and expansion of seasonal malaria chemoprevention (SMC) among U5 s [12] has the potential to influence caregiver recall of anti-malarial drugs used for curative purposes. Seasonal malaria chemoprevention aims to reduce malaria morbidity and mortality in children under five in high transmission settings by administration of SP and amodiaquine to children aged 3–59 months at 1-month intervals for up to 4 months during the peak transmission season [13]. Patients in Mali often seek treatment from a combination of public and private sector locations [10], and while policies are in place to provide free malaria diagnosis and treatment in the public sector, public health facilities operate on a cost-recovery basis and are dependent on user fees and pharmaceutical sales [14]. Literacy rates among Malian women are low [10], and a previous study identified inconsistencies in local language terms for specific anti-malarial medicines [15].

This study aimed to assess the validity of malaria diagnosis and treatment coverage indicators collected during household surveys. Specifically, the validity of caregiver-reported receipt of anti-malarials for febrile children seeking treatment from community health workers (CHWs), public facilities in rural and urban areas, and private facilities in urban areas both prior to and during the SMC campaign period were ascertained.

## Methods

### Study site

The study was conducted in urban areas of Bamako and Sikasso, and in rural areas of Niena District, Sikasso region. Bamako has a population of approximately 2.3 million, while the population of Sikasso town is approximately 600,000 and Niena district 160,000. Niena district and Sikasso town are in the Sudano-Guinean zone of high malaria transmission, while Bamako is in the Sahelian zone and experiences lower levels of malaria transmission due to the urban environment [16]. The Sudano-Guinean zone experiences perennial malaria transmission with a seasonal peak during June–November, while the Sahelian zone has a shorter transmission season and similar seasonal peak.

Mali adopted ACT as treatment for uncomplicated malaria in 2005, replacing chloroquine [17]. At present, artemether–lumefantrine is the recommended first-line treatment for uncomplicated malaria, with artesunate–amodiaquine as the second-line. National malaria treatment guidance recommends that children with severe malaria receive an injection of artesunate, artemether or quinine (according to availability), followed by a full dose of ACT as soon as they are able to ingest the drug without vomiting [17]. Laboratory diagnosis (by microscopy or RDT) and treatment of malaria is free at all levels of the health system for U5s and pregnant women.

Mali introduced a policy of SMC in 2012, with implementation commencing in 2014. Nationwide SMC was conducted for the first time in 2016, implemented door-to-door using a dispersible format of co-blistered SP and amodiaquine. During the SMC campaign, any child eligible for SMC who has a current or reported fever was tested by RDT. Children with a positive RDT and no signs of severe disease received ACT in accordance with standard treatment guidelines, and did not receive SMC drugs. Coverage of SMC was high across all study areas in 2017: 88–109% by round in urban Sikasso, 96–103% by round in Niena district, and 112–124% by round in Bamako (S. Fomba, personal communication). Coverage exceeding 100% is a result of the number of children receiving SMC exceeding the estimated eligible population.

**Fig. 1:**
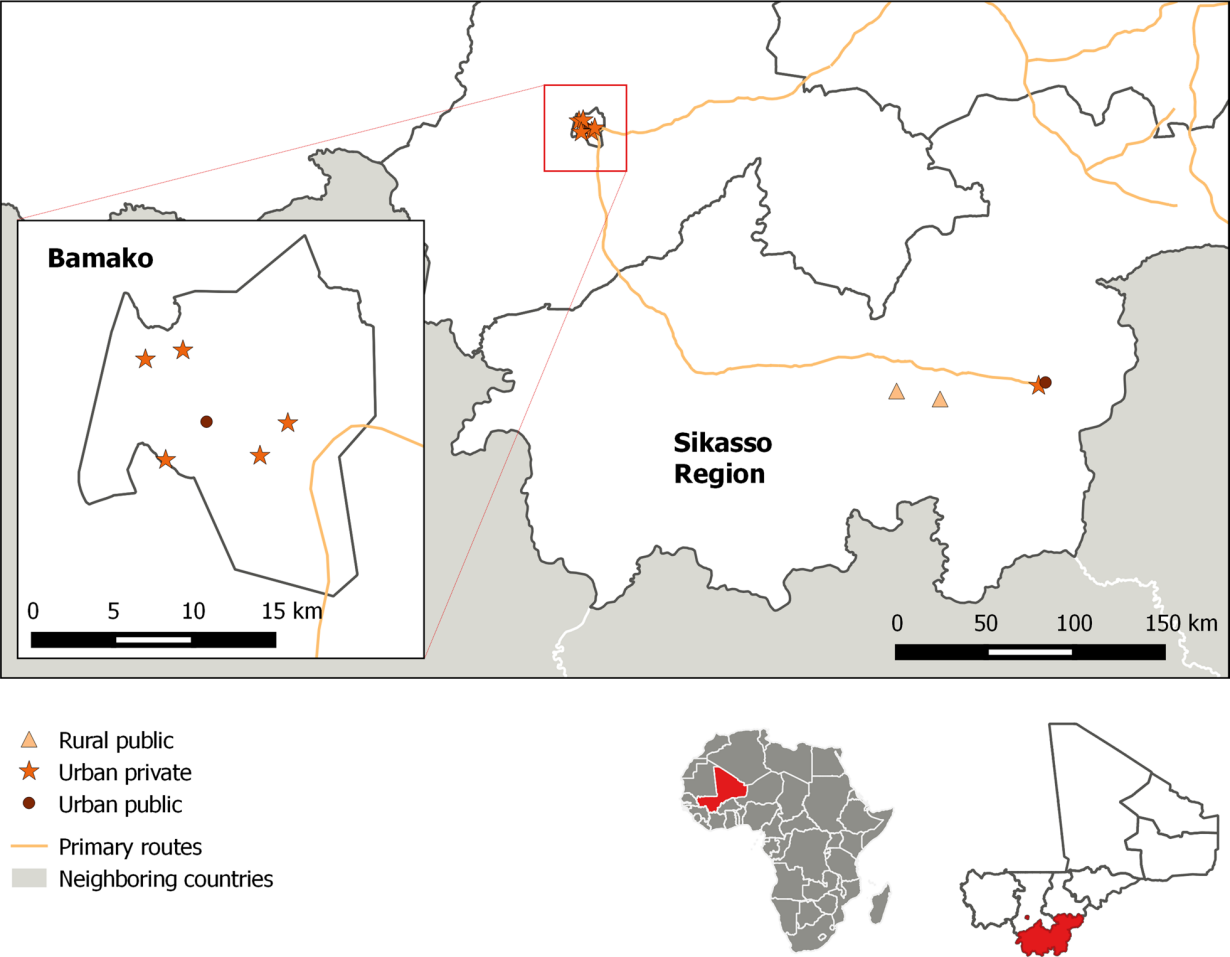
Map of participating public urban facilities, public rural facilities, and private urban facilities. Participating community health workers were close to the two public rural facilities

Four different types of health care services were included in the study: urban private health facilities, urban public health facilities, rural public health facilities and CHWs. Public health facilities were purposively selected for inclusion in the study based on their weekly patient flow and mean weekly number of confirmed malaria cases in children U5 during the 2016 malaria transmission season. No database capturing the number of malaria cases recorded in private health facilities exists, therefore, private facilities were identified using local knowledge about facilities with high attendance by children under 5 years. Initially, two public urban facilities [one district referral health center (CSRef) in Sikasso, one community health centre (CSCom) in Bamako], two public rural facilities (CSComs) in Niena district, eight CHWs working in the catchment area of the two rural CSComs, and two private facilities in Bamako were selected for participation. Four additional private urban facilities were added due to slow recruitment of participants in the first weeks of the study, with only the two highest-recruiting private facilities retained for the second half of the study. One additional CHW in the same Niena catchment area was added halfway through the study (Fig. 1).

### Study design, participants and data collection procedures

A prospective unmatched case control study design was used to assess the validity of primary coverage indicators for diagnosis and treatment of malaria in children U5, as estimated in household surveys. Caregivers aged at least 18 years attending study health facilities or CHWs with a current/recently febrile child U5 were eligible to be recruited to the study. Data collection was divided into two periods: July 2017, prior to the start of the SMC campaign; and September–November 2017, concurrent with the SMC campaign.

For the ACT coverage indicator, ‘cases’ were defined as caregivers of children aged one to 59 months (U5) who were prescribed ACT at the health facility or CHW site, while ‘controls’ were caregivers of children U5 who attended the health facility or CHW site, but were not prescribed ACT. Children with signs of severe malaria or other serious illness were excluded.

Details of the child’s consultation, including diagnostic used (if any), test result, final diagnostic decision of health worker, and drugs prescribed were recorded by the health worker during or immediately after the consultation using simple data collection forms. These served as the ‘gold standard’ of procedures and drugs during the consultation [18]. Health workers were told the general aim of the study, but advised to continue their consultation and prescribing practices as normal and not to divulge the specific aim of the study to participants.

Eligible caregivers were asked by the health worker if they would like to be involved in a study related to child health, and interested caregivers were consented and registered by the study team at the health facility (or by the CHW at CHW sites). Caregivers provided basic information to allow a follow-up interview at their home. Follow-up interviews were randomised to be completed either 1–7 or 8–14 days following the consultation.

Informed consent was obtained from caregivers when visited at their home for the follow-up interview. The interview was directed to the caregiver who attended the facility or CHW site with the child, and used a standardised questionnaire based on the DHS relating to recent fever and treatment-seeking for children U5 years. The questionnaire also included standard DHS questions relating to caregiver’s sex, age, literacy, and education level, as well as ownership of household assets. At the end of the questionnaire, further detail on treatment received was collected using a visual aid with photographs of common drugs prescribed for fever in Mali (Additional file 1), and requesting to review documents or packaging retained by the caregiver from the consultation.

All data were collected on paper-based forms, then double-entered into a CSPro template and discrepancies resolved by reviewing original questionnaires. Drug names and brands reported by caregivers that were not pre-coded in the questionnaire were classified by first searching the ACTwatch database of anti-malarial drugs [19], then using broader internet searches if not found in the anti-malarial database.

### Sample size

A sample size of 200 cases (received ACT) and 200 controls (did not receive ACT) were required for each of the four types of recruitment site (public urban, public rural, private urban and CHW). The sample size calculation assumed a 5% probability of committing a type-1 error (2-tailed, sensitivity of 60–70%, specificity of 70–80%, precision of ± 7%, and interview refusal rate of 10%. The total required sample size was 1600, split evenly between the periods before and during the SMC campaign.

### Statistical analysis

Data management and cleaning were performed with Stata version 14. Primary outcomes for this study were the sensitivity, specificity and accuracy of caregivers’ recall of the child having received a finger/heel prick test for malaria, result of the malaria test, final diagnosis made by the health worker, and treatment received.

Additional outcomes of corrected ACT recall were created, where the caregivers’ recall of ACT was defined as reporting during the interview that ACT was received for the child, identifying ACT on the visual aid, having a prescription document listing ACT received by the child, or having retained ACT packaging from the child’s recent illness. These “corrected ACT recall” variables were generated for each correction method separately (visual aid, prescription, or retained packaging) and where ACT was reported by interview or any of the correction methods.

Household socio-economic status was derived using principal components analysis of household assets, then divided into wealth quintiles [20]. Both binary assets (e.g. radio ownership) and continuous variables (number of cows, sheep, etc.) were included in the principal components analysis. Assets with ownership frequency < 5% were excluded.

Sensitivity, specificity and accuracy and their respective 95% confidence intervals (CIs) were estimated using the Huber–White Sandwich estimator to account for correlated data within each health facility or CHW site [21]. Sensitivity represents the proportion of caregivers who correctly recalled that the child received ACT; specificity is the proportion of caregivers who correctly recalled that the child did not receive ACT; and accuracy represents the proportion of caregivers whose recall of whether ACT was received by the child agreed with the gold standard records made by the clinician during the consultation. Differences in sensitivity, specificity and accuracy by caregiver and child characteristics were assessed using a Chi squared test, or by Fishers’ exact test where any cell value was below 10.

Logistic regression models were used to estimate adjusted odds ratios for facility, caregiver and child characteristics associated with sensitivity, specificity and accuracy of ACT recall. Sensitivity, specificity or accuracy of ACT recall were included as primary binary outcomes in three separate models, with a further set of models generated for corrected ACT recall (recall including visual aids, review of prescription and retained packaging). The sensitivity model binary outcome variable was 1 when caregivers correctly reported that the child received ACT, and 0 when caregivers incorrectly reported that the child did not receive ACT when they did. The specificity model binary outcome variable was 1 when caregivers correctly reported that the child did not received ACT, and 0 when the caregiver incorrectly reported that the child received ACT when they did not. The accuracy binary outcome variable was 1 when caregivers correctly reported if the child did or did not received ACT, and 0 when the caregiver incorrectly reported if the child did or did not receive ACT. The models developed included site of enrolment as a random intercept, with all other variables of interest included as fixed effects.

Individual-level validity (of recall of test, test result, diagnosis, treatment) was estimated by calculating the area under the receiver operator curve (AUC) for plots of sensitivity against 1-specificity for all sites combined, and for each type of site individually [18]. Specific treatment recall variables were recall of any anti-malarial treatment, recall of ACT, and corrected recall of ACT.

Population-level validity of recall was assessed by estimating an inflation factor (IF), which gives an estimate of the extent to which the survey-based estimates provide an unbiased estimate of the true coverage [18]. Briefly, using measured sensitivity and specificity of test, test result, diagnosis and treatment recall, the coverage that would be measured by a household survey is modelled across a range of ‘gold standard’ coverage levels. Stata code produced by Munos et al. was used to prepare IF plots [18].

## Results

**Fig. 2:**
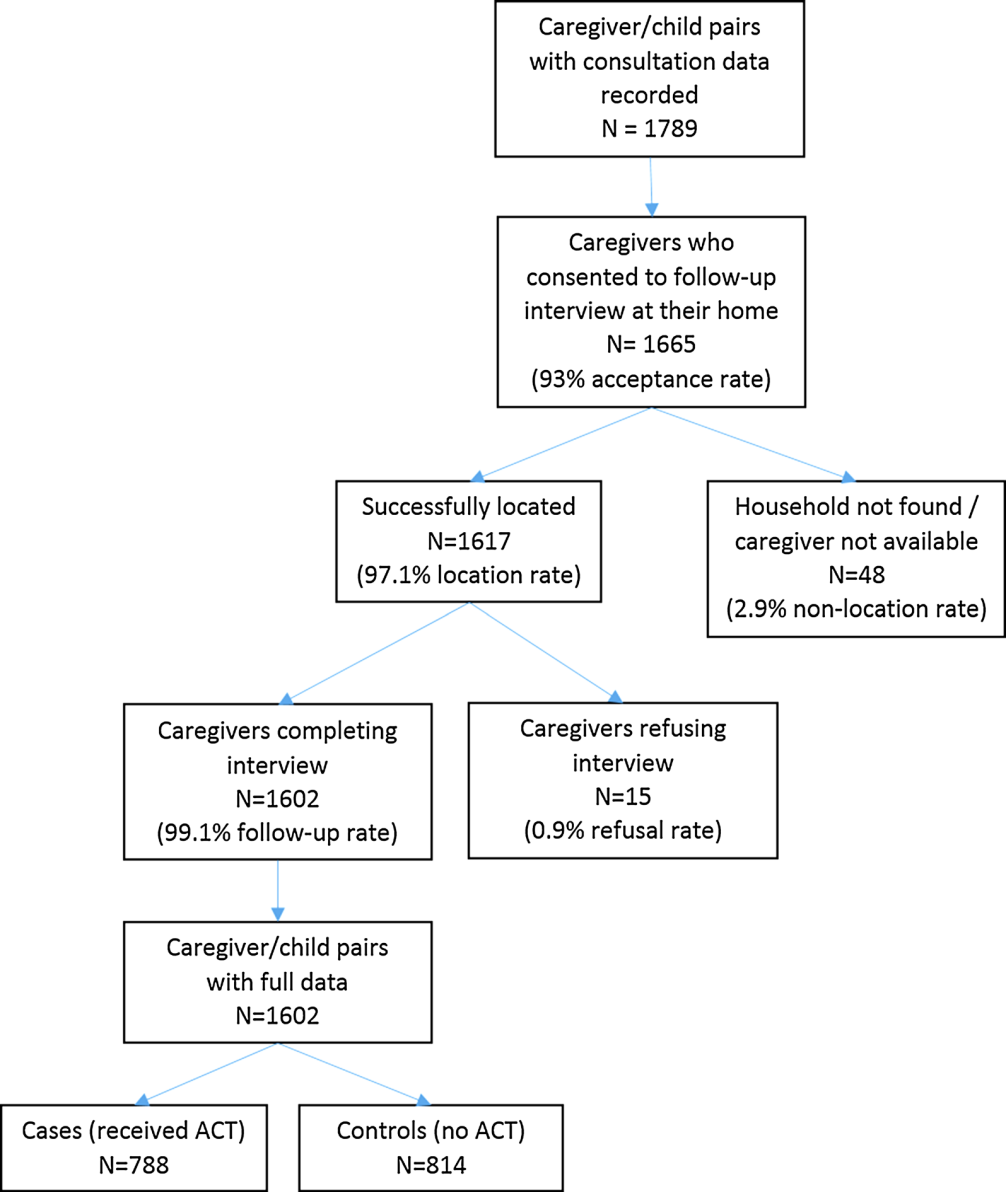
Participant enrolment flowchart

A total of 1789 caregivers were approached to participate in the study, with 1665 (93%) accepting. Follow-up interviews were successfully completed with 1602 caregivers, with 49% being caregivers of children who received ACT during the consultation (Fig. 2). Participant enrolment over time is described further in Additional file 2. Only 38.0% of participants were recruited in July, prior to the start of the SMC campaign, lower than the 50% target.

The majority (82%) of enrolled children were taken to the health facility by a female caregiver, but the proportion varied by type of facility (p < 0.001, Table 1). Overall, 46.9% of caregivers had not attended formal education, and 58.1% could not read. Both these characteristics showed considerable variation by type of health facility (p < 0.001). Slightly more male children were recruited than female children, with no evidence for difference by type of facility.

**Table 1:** Demographics of enrolled children and attending caregivers who completed both registration at health facility and follow-up interview

	All cases (received ACT)		All controls (no ACT)		p-value	Pooled data for cases and controls										
						All sites combined		Public Urban		Public Rural		CHW		Private Urban		p-value
	n	%	n	%		n	%	n	%	n	%	n	%	n	%	
Number of child/caregiver pairs																
Pre-SMC	315	40.0	294	36.1		609	38.0	196	46.6	220	52.4	115	27.1	78	23.0	
During SMC	473	60.0	520	63.9	0.112	993	62.0	225	53.4	200	47.6	310	72.9	258	76.8	< 0.001
Sex of sick child																
Male	415	52.7	457	56.1		872	54.4	228	54.2	236	56.2	222	52.2	186	55.4	
Female	373	47.3	357	43.9	0.162	730	45.6	193	45.8	184	43.8	203	47.8	150	44.6	0.687
Age of sick child in years																
< 1	108	13.7	197	24.2		305	19.0	69	16.4	76	18.1	100	23.5	60	17.9	
1	175	22.2	154	18.9		329	20.5	107	25.4	75	17.9	55	12.9	92	27.4	
2	186	23.6	181	22.2		367	22.9	103	24.5	84	20.0	99	23.3	81	24.1	
3	147	18.7	140	17.2		287	17.9	70	16.6	94	22.4	61	14.4	62	18.5	
4	172	21.8	142	17.4	< 0.001	314	19.6	72	17.1	91	21.7	110	25.9	41	12.2	< 0.001
Period between consultation and interview																
1–7 days	486	61.7	513	63.0		999	62.4	254	60.3	270	64.3	266	62.6	209	62.2	
8–14 days	302	38.3	301	37.0	0.578	603	37.6	167	39.7	150	35.7	159	37.4	127	37.8	0.702
Sex of caregiver																
Male	157	19.9	132	16.2		289	18.0	82	19.5	128	30.5	24	5.6	55	16.4	
Female	631	80.1	682	83.8	0.054	1313	82.0	339	80.5	292	69.5	401	94.4	281	83.6	< 0.001
Caregiver age in years																
18–24	196	24.9	220	27.0		416	26.0	112	26.6	118	28.1	113	26.6	73	21.7	
25–34	367	46.6	375	46.1		742	46.3	200	47.5	174	41.4	203	47.8	165	49.1	
35–44	161	20.4	169	20.8		330	20.6	83	19.7	83	19.8	78	18.4	86	25.6	
≥ 45	64	8.1	50	6.1	0.397	114	7.1	26	6.2	45	10.7	31	7.3	12	3.6	0.002
Caregiver education level																
None	358	45.4	393	48.4		751	46.9	102	24.2	280	66.8	341	80.4	28	8.3	
Primary	196	24.9	186	22.9		382	23.9	118	28.0	122	29.1	77	18.2	65	19.3	
Secondary or higher	234	29.7	233	28.7	0.464	467	29.2	201	47.7	17	4.1	6	1.4	243	72.3	< 0.001
Literacy of caregiver																
Cannot read	409	56.9	422	59.4		831	58.1	114	30.8	316	75.4	364	85.6	37	17.1	
Can read part of test sentence	73	10.2	78	11.0		151	10.6	44	11.9	46	11.0	38	8.9	23	10.6	
Read all of test sentence	229	31.9	204	28.7		433	30.3	197	53.2	57	13.6	23	5.4	156	72.2	
No card in appropriate language	8	1.1	7	1.0	0.607	15	1.0	15	4.1	0	0.0	0	0.0	0	0.0	< 0.001
Wealth index of caregiver																
Poorest	163	21.1	153	19.1		316	20.1	3	0.7	160	38.9	153	36.6	0	0.0	
2nd	145	18.8	170	21.2		315	20.0	27	6.5	128	31.1	153	36.6	7	2.1	
Middle	164	21.2	151	18.8		315	20.0	79	18.9	98	23.8	103	24.6	35	10.6	
4th	174	22.5	141	17.6		315	20.0	177	42.4	23	5.6	9	2.2	106	32.1	
Wealthiest	127	16.4	188	23.4	0.002	315	20.0	131	31.4	2	0.5	0	0.0	182	55.2	< 0.001

Across all sites, half (50%) of children received a confirmatory diagnostic test for malaria, and 73% of those tested had a positive test result (Table 2). Irrespective of test results, 76% of all participating children received a diagnosis of malaria from the health worker, 49% received ACT, and 79% received an anti-malarial drug of any type. Non-ACT anti-malarials prescribed were primarily injectable artesunate, but also included artemether, amodiaquine and quinine. Health worker diagnostic and prescription practices reported during this study will be presented in detail in a separate publication.

**Table 2:** Summary of malaria diagnostic procedures and treatment prescribed at participating health facilities and community health worker (CHW) sites, as recorded by the health worker during or immediately after consultation

	All cases (received ACT)		All controls (no ACT)		Pooled data for cases and controls									
					All sites combined		Public Urban		Public Rural		CHW		Private Urban	
	n	%	n	%	n	%	n	%	n	%	n	%	n	%
Diagnostic method (N = 1601)														
No diagnostic method used	5	0.6	19	2.3	24	1.5	0	0.0	0	0.0	0	0.0	24	7.1
Clinical signs/symptoms only	334	42.4	449	55.2	783	48.9	69	16.4	306	73.0	142	33.4	266	79.2
Rapid diagnostic test	299	38.0	208	25.6	507	31.7	101	24.0	112	26.7	283	66.6	11	3.3
Microscopy	149	18.9	138	17.0	287	17.9	251	59.6	1	0.2	0	0.0	35	10.4
Temperature measured (N = 1602)														
No	44	5.6	48	5.9	92	5.7	37	8.8	18	4.3	20	4.7	17	5.1
Yes	744	94.4	766	94.1	1510	94.3	384	91.2	402	95.7	405	95.3	319	94.9
Diagnostic test result (N = 1602)														
Negative for malaria	80	10.2	135	16.6	215	13.4	88	20.9	14	3.3	101	23.8	12	3.6
Positive for malaria	365	46.3	208	25.6	573	35.8	264	62.7	99	23.6	179	42.1	31	9.2
Not tested	333	42.3	464	57.0	797	49.8	69	16.4	303	72.1	135	31.8	290	86.3
Invalid	8	1.0	4	0.5	12	0.7	0	0.0	3	0.7	9	2.1	0	0.0
Don’t know	2	0.3	3	0.4	5	0.3	0	0.0	1	0.2	1	0.2	3	0.9
Health worker final diagnosis (N = 1559)														
Not malaria	56	7.1	326	40.2	382	23.9	74	17.6	24	5.7	136	32.2	148	44.0
Malaria	731	98.9	486	59.9	1217	76.1	347	82.4	396	94.3	286	67.8	188	56.0
Treatment provided^a^ (N = 1602)														
ACT	788	100	0	0	788	49.2	231	54.9	205	48.8	212	49.9	140	41.7
Non-ACT anti-malarial^b^	262	33.3	472	58.1	734	45.8	184	43.7	374	89.0	111	26.1	65	19.4
ACT + non-ACT anti-malarial	262	33.3	0	0	262	16.4	41	9.7	176	41.9	27	6.4	18	5.4
Antipyretics	653	82.9	641	78.8	1294	80.8	322	76.5	414	98.6	387	91.1	171	50.9
Antibiotics	499	63.3	531	65.3	1030	64.3	323	76.7	366	87.1	147	34.6	194	57.7
Other drugs^c^	278	35.3	323	39.7	601	37.5	151	35.9	251	59.8	78	18.4	121	36.0
No drugs	0	0	1	0.1	1	0.1	0	0.0	0	0.0	0	0.0	1	0.3
Where caregiver intends to collect drugs (N = 1593)														
The facility/CHW	454	58.0	513	63.3	967	60.7	145	34.4	418	99.5	396	94.7	8	2.4
Another location	253	32.3	128	15.8	381	23.9	257	61.0	1	0.2	16	3.8	107	32.0
Depends on patient preference	68	8.7	161	19.9	229	14.4	15	3.6	0	0.0	0	0.0	214	64.1
Don’t know	8	1.0	8	1.0	16	1.0	4	1.0	1	0.2	6	1.4	5	1.5

*ACT* artemisinin-based combination therapy, *CHW* community health worker

^a^More than one drug may have been prescribed, numbers do not sum to 100%

^b^Includes artemether, artesunate, amodiaquine, quinine

^c^Includes antiemetic, antifungal, antihistamine, anthelmintic, bronchodilator/mucolytic, intravenous infusion, multivitamin, steroid, zinc/oral rehydration salts

Table 3 presents the sensitivity, specificity and accuracy of caregiver’s recall of diagnosis and treatment the child received during the consultation, against the gold standard recorded by the health worker. Caregivers’ recall of a finger/heel prick and a malaria test result were good; there was a high sensitivity and specificity of recall of a finger/heel prick (91.5% and 85.7%, respectively), and high sensitivity but moderate specificity for recall of a positive test result (96.2% and 59.7%, respectively). Caregivers’ recall of a malaria diagnosis being made by the health worker had moderate sensitivity and specificity (74.3% and 74.9%, respectively). Caregivers’ recall of treatment had low sensitivity for both any anti-malarial (59.0%) and ACT (43.2%), however specificity was high for both (82.7% and 90.2, respectively). Documents describing drugs provided to the child had been retained by 87.6% of caregivers, and were available for review by the interviewer among 82.2% of these individuals. Drug packaging had been retained by 87.4% of caregivers, with 98.8% of these caregivers able to show the drug packet to the interviewer. Sensitivity of ACT recall was dramatically increased by incorporating identification of ACT from visual aids, prescriptions or retained packaging (91.5%), but with a slight reduction in specificity to 71.7%. Sensitivity, specificity and accuracy of recall of diagnosis and treatment are presented by facility type in Additional file 3.

**Table 3:** Sensitivity, specificity and accuracy of caregivers’ recall of diagnosis procedures at facilities and treatment received during consultation, summarized for all facility types

	Sensitivity^a^			Specificity^b^			Accuracy^c^		
	N	%	95% CI	N	%	95% CI	N	%	95% CI
Recall of fever in past 2 weeks	1602	99.7	99.4, 100.0	0	–	–	1602	99.7	99.4, 100.0
Recall of finger/heel stick	792	91.5	89.6, 93.5	796	85.7	83.2, 88.1	1588	88.6	87.0, 90.2
Recall of positive malaria test result^d^	421	96.2	94.4, 98.0	154	59.7	51.9, 67.6	575	86.4	83.6, 89.2
Recall that malaria diagnosis was made	1213	74.3	71.8, 76.7	372	74.9	70.5, 79.3	1588	74.4	72.3, 76.6
Recall of any anti-malarial given^e^	1235	59.0	56.3, 61.8	336	82.7	78.7, 86.8	1571	64.1	61.7, 66.5
Recall of ACT given^e^	755	43.2	39.6, 46.7	794	90.2	88.1, 92.3	1549	67.3	64.9, 69.6
Corrected recall of ACT given^f^	787	91.5	89.5, 93.4	807	71.7	68.6, 74.9	1594	81.5	79.6, 83.4

*ACT* Artemisinin-based combination therapy

^a^Sensitivity calculated as total true positives (not shown) divided by the number of true positives and false negatives (N)

^b^Specificity calculated as the total true negatives (not shown) divided by the number of true negatives and false positives (N)

^c^Accuracy calculated as the sum of true positives and true negatives (not shown) divided by sum of true positives, true negatives, false positives and true negatives (N)

^d^Among those children tested

^e^Excludes treatment that caregiver reported receiving from a different facility or healthcare provider

^f^Recall of ACT from interview corrected to include instances where caregiver identified ACT from the visual aid, or had retained a prescription including ACT or ACT packaging from the child’s consultation

In multivariate models, sensitivity of ACT recall was higher at CHW sites than in the reference group of public urban facilities (adjusted odds ratio [AOR] of 12.2), with no significant differences between other site types (Table 4). Sensitivity of recall was also significantly lower among caregivers from the two poorest quintiles than caregivers from the richest quintile (AOR = 0.20 and AOR = 0.37, respectively). Literacy was associated with sensitivity of ACT recall, with those who were illiterate or could read only part of a test sentence having lower sensitivity than literate caregivers (AOR 0.27 and AOR 0.38, respectively). Specificity of ACT recall differed by study period, with lower specificity during the period of SMC implementation compared with the period before SMC campaigns began (AOR 0.33). When reviewing the multivariate model for “corrected ACT recall” which included caregiver identification of ACTs from visual aids, prescriptions or retained packaging (Additional file 4), attending a CHW site was no longer significantly associated with recall sensitivity (p = 0.077), however the associations between recall sensitivity and caregiver literacy (AOR = 0.29, p = 0.006 comparing those who can read only a little against those who were literate) and between specificity and SMC were retained (AOR 0.53, p = 0.003). Summaries of sensitivity, specificity and accuracy of ACT recall by various facility, caregiver and child characteristics, together with Chi squared test p-values are presented in Additional file 5, with summaries for ACT recall corrected by visual aid, prescription or packaging in Additional file 6.

**Table 4:** Random effects multivariate logistic regression models of sensitivity, specificity and accuracy of caregivers’ recall of treatment with artemisinin-based combination therapy (ACT): associations with follow-up and socio-demographic characteristics

	Sensitivity		Specificity		Accuracy	
	AOR (95% CI)	p-value	AOR (95% CI)	p-value	AOR (95% CI)	p-value
Type of facility						
Public urban	1.00		1.00		1.00	
Public rural	5.44 (0.40, 74.0)	0.203	0.72 (0.25, 2.07)	0.539	2.57 (0.72, 9.14)	0.144
CHW	12.21 (1.33, 111.75)	0.027	2.22 (0.70, 7.08)	0.176	5.00 (1.66, 15.03)	0.004
Private urban	0.53 (0.06, 4.81)	0.573	0.94 (0.30, 2.95)	0.912	0.32 (0.10, 1.01)	0.051
Region						
Bamako	1.08 (0.12, 9.95)	0.945	3.42 (1.10, 10.63)	0.033	3.74 (1.19, 11.74)	0.024
Sikasso	1.00		1.00		1.00	
Time period						
Before SMC	1.00		1.00		1.00	
During SMC	1.24 (0.84, 1.85)	0.278	0.33 (0.17, 0.53)	0.001	0.91 (0.70, 1.18)	0.469
Child’s age in years						
< 1	0.90 (0.46, 1.74)	0.753	1.00 (0.39, 2.57)	0.992	1.47 (1.11, 2.54)	0.015
1	0.88 (0.49, 1.56)	0.652	0.61 (0.24, 1.58)	0.309	1.03 (0.70, 1.52)	0.880
2	1.28 (0.75, 2.19)	0.371	0.64 (0.26, 1.55)	0.320	1.10 (0.76, 1.59)	0.607
3	0.99 (0.55, 1.76)	0.966	1.02 (0.38, 2.71)	0.966	1.39 (0.94, 2.07)	0.103
4	1.00		1.00		1.00	
Child’s sex						
Male	0.84 (0.59, 1.21)	0.354	0.75 (0.43, 1.31)	0.312	0.95 (0.75, 1.21)	0.685
Female	1.00		1.00		1.00	
Days to follow-up						
1–7	1.00		1.00		1.00	
8–14	1.00 (0.69, 1.45)	0.992	0.83 (0.48, 1.43)	0.506	0.91 (0.71, 1.18)	0.484
Caregiver’s sex						
Male	0.87 (0.52, 1.47)	0.614	1.43 (0.61, 3.33)	0.407	1.07 (0.75, 1.52)	0.721
Female	1.00		1.00		1.00	
Caregiver’s age						
18–24	1.17 (0.54, 2.55)	0.698	1.88 (0.53, 6.59)	0.325	1.64 (0.97, 2.76)	0.063
25–34	1.23 (0.60, 2.52)	0.579	1.23 (0.38, 3.96)	0.734	1.42 (0.88, 2.29)	0.154
35–44	1.99 (0.91, 4.27)	0.079	2.11 (0.57, 7.78)	0.260	1.83 (1.09, 3.06)	0.022
≥ 45	1.00		1.00		1.00	
Wealth index of caregiver						
Poorest	0.20 (0.08, 0.48)	< 0.001	3.83 (1.02, 14.41)	0.047	0.66 (0.36, 1.18)	0.158
2nd	0.37 (0.16, 0.87)	0.023	3.54 (1.00, 12.47)	0.049	1.02 (0.58, 1.80)	0.939
Middle	0.72 (0.34, 1.51)	0.383	2.77 (0.89, 8.60)	0.077	1.07 (0.65, 1.76)	0.805
4th	0.74 (0.40, 1.37)	0.337	2.15 (0.82, 5.89)	0.121	0.89 (0.59, 1.36)	0.605
Wealthiest	1.00		1.00		1.00	
Literacy of caregiver						
Cannot read at all	0.27 (0.16, 0.46)	< 0.001	0.77 (0.35, 1.69)	0.509	0.54 (0.38, 0.77)	0.001
Can read a little	0.38 (0.19, 0.77)	0.007	2.04 (0.53, 7.94)	0.302	0.71 (0.45, 1.14)	0.159
Can read all of sentence	1.00		1.00		1.00	

*AOR* adjusted odds ratios, *CHW* community health worker, *SMC* seasonal malaria chemoprevention

**Fig. 3:**
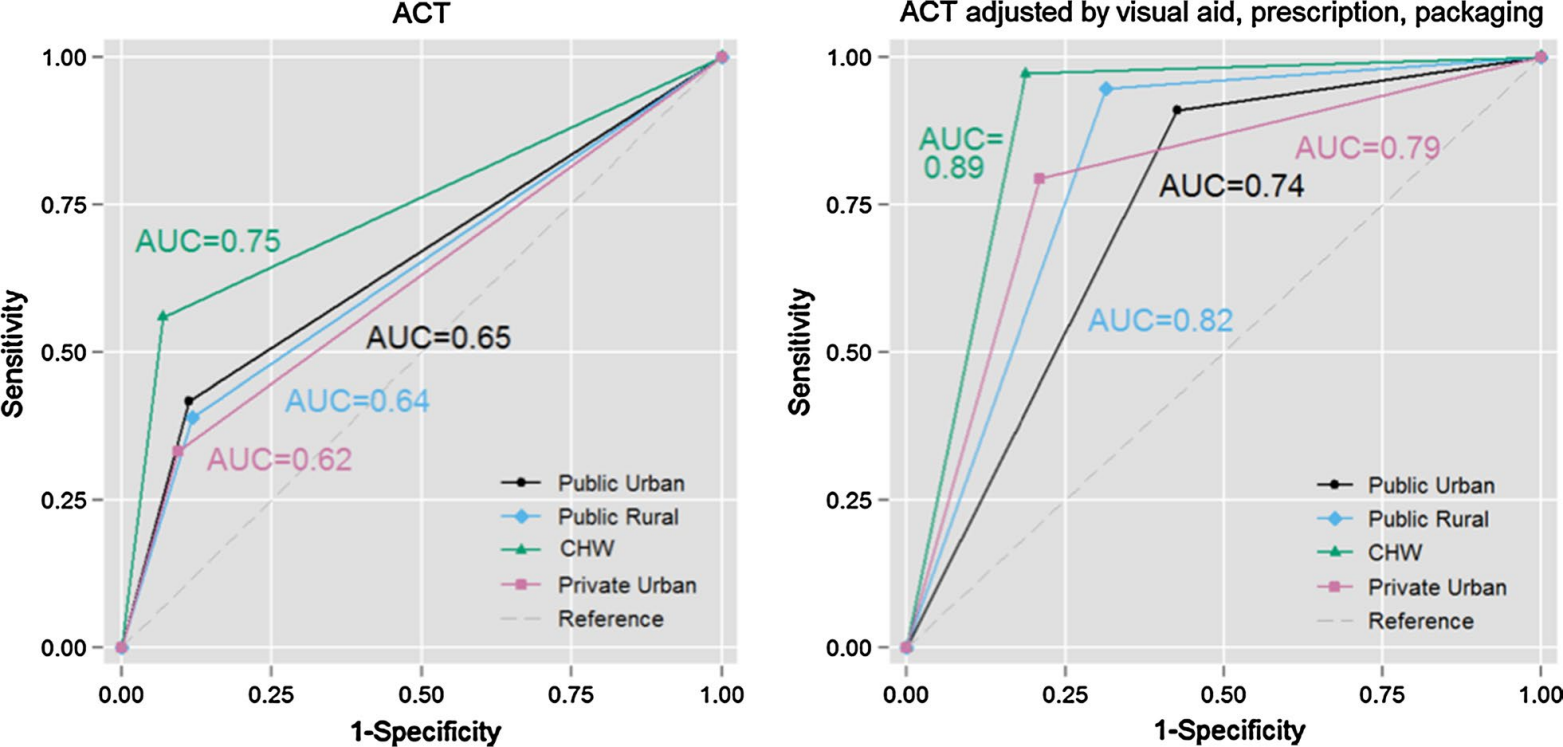
Receiver operating characteristic (ROC) curve by site for recall of artemisinin-based combination therapy (ACT), and ROC by site type for corrected recall of ACT

Individual-level validity of diagnosis and treatment recall (defined by those that correctly recalled the event in question) is estimated using AUC (Table 5, Fig. 3). Recall of a finger/heel stick, a positive malaria test, and a malaria diagnosis by the health worker all had AUC > 0.7, indicating good individual-level validity, with some variation by facility type. Caregivers’ recall of ACT being provided to the child had only moderate individual-level validity at all site types except at CHW sites, where AUC was 0.75. Including visual aids, prescription and packaging review in ACT recall increased AUC > 0.7 at all site types (Additional files 7, 8).

**Table 5:** Area under the curve (AUC) of the receiver operating characteristic (ROC) including 95% confidence intervals for each of the main recall indicators, across all sites and by type of site

	All sites		Public urban		Public rural		CHWs		Private urban	
	AUC	95% CI	AUC	95% CI	AUC	95% CI	AUC	95% CI	AUC	95% CI
Recall of finger/heel stick	0.89	0.87, 0.90	0.86	0.81, 0.91	0.80	0.75, 0.84	0.82	0.78, 0.86	0.96	0.93, 0.98
Recall of positive malaria test result^a^	0.78	0.74, 0.82	0.73	0.67, 0.79	0.74	0.24, 1.00	0.81	0.75, 0.87	0.90	0.78, 1.00
Recall that malaria diagnosis was made	0.75	0.72, 0.77	0.75	0.70, 0.81	0.60	0.50, 0.71	0.74	0.70, 0.79	0.81	0.77, 0.85
Recall of any anti-malarial given^b^	0.70	0.68, 0.73	0.68	0.61, 0.75	0.62	0.50, 0.74	0.68	0.64, 0.73	0.72	0.68, 0.77
Recall of ACT given^b^	0.67	0.65, 0.69	0.65	0.61, 0.69	0.64	0.60, 0.68	0.75	0.71, 0.78	0.62	0.57, 0.66
Recall of ACT, corrected by visual aid	0.74	0.72, 0.76	0.69	0.65, 0.74	0.74	0.69, 0.78	0.82	0.79, 0.86	0.69	0.64, 0.73
Recall of ACT, corrected by retained prescription	0.79	0.77, 0.81	0.76	0.72, 0.80	0.80	0.77, 0.84	0.81	0.77, 0.85	0.75	0.71, 0.80
Recall of ACT, corrected by retained packaging	0.81	0.79, 0.83	0.74	0.69, 0.78	0.80	0.77, 0.84	0.89	0.86, 0.92	0.78	0.73, 0.82
Recall of ACT, corrected by visual aid, prescription or retained packaging	0.82	0.80, 0.83	0.74	0.70, 0.78	0.82	0.78, 0.85	0.89	0.86, 0.92	0.79	0.75, 0.84

A high individual-level accuracy is generally considered 13 to be AUC > 0.7; moderate individual-level accuracy to be AUC 0.6–0.7; and low individual-level accuracy AUC < 0.6, based on recommendations from the Child Health Epidemiology Reference Group (CHERG) and Improving Coverage Measurement (ICM) group

*ACT* artemisinin-based combination therapy, *AUC* area under [ROC] curve, *CHW* community health worker

^a^Among those children tested

^b^Excludes treatment that caregiver reported receiving from a different facility or healthcare provider

**Fig. 4:**
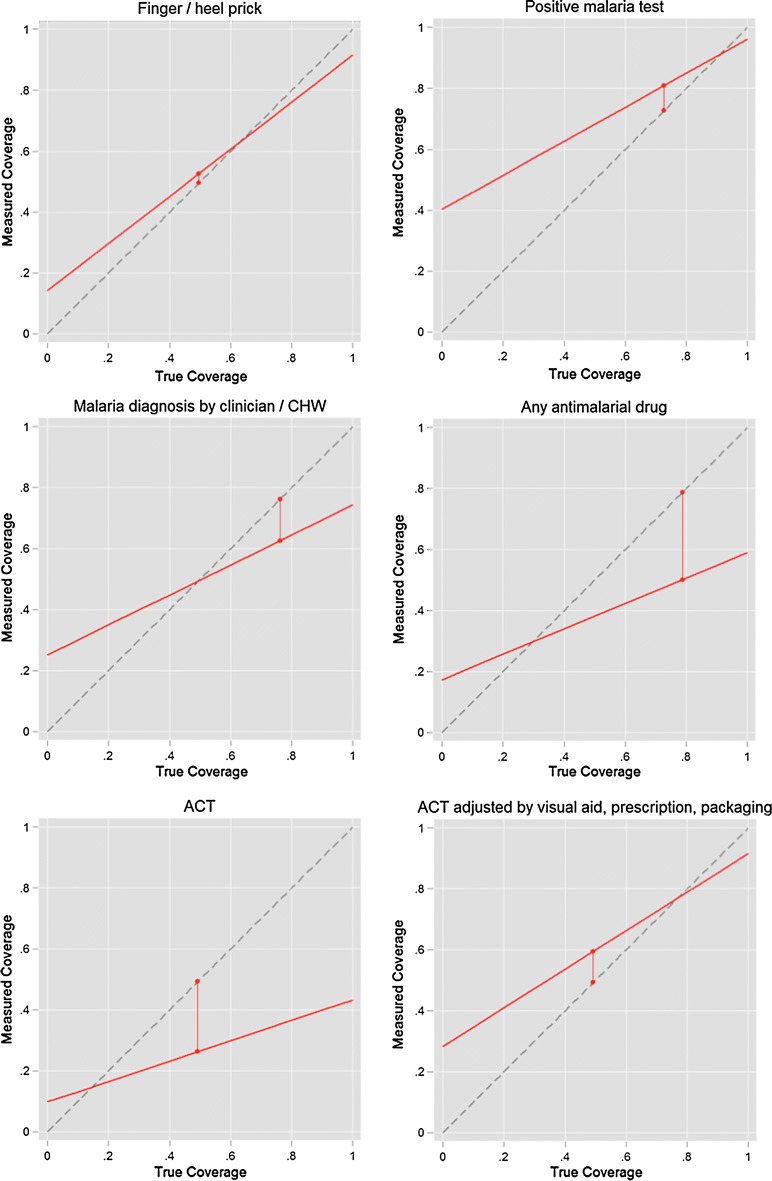
Inflation factor scatterplots for (i) blood test; (ii) positive blood test; (iii) malaria diagnosis (clinical or confirmed); (iv) receipt of any anti-malarial; (v) receipt of artemisinin-based combination therapy (ACT); (vi) receipt of ACT, corrected by visual aid, prescription review or retained packaging review

Table 6 shows modelled estimates of the inflation factor for recall of each of the diagnosis and treatment indicators. Population-level bias is low (0.75–1.25) for recall of a finger/heel prick and a positive malaria test at all site types. Population-level bias was moderate to high (0.47 to 0.62 by site type) for recall of ACT, however bias was reduced when modelling corrected recall (including ACT identified by visual aid, retained packaging or prescription). Modelled estimates of coverage of each recall indicator for a range of coverage levels are shown in Fig. 4. Sensitivity and specificity of caregivers’ recall of a positive malaria test indicates that this indicator would likely be over-estimated at low levels of parasitological testing, but is accurate when a parasitological testing rates are high. Coverage of ACT is likely to be increasingly underestimated at higher levels of ACT coverage, but is estimated to be more accurate where fewer children receive ACT when seeking treatment for fever. When ACT recall is corrected using visual aids, prescription and packaging review, modelled estimates of ACT coverage are a closer match to true coverage across a wider range of ACT coverage levels.

**Table 6:** Inflation factor (IF) including 95% confidence intervals for each of the main recall indicators, across all sites and by type of site

	All sites	Public urban	Public rural	CHWs	Private urban
Recall of finger/heel stick	1.06	1.01	1.12	1.05	1.35
Recall of positive malaria test result^a^	1.11	1.11	1.05	1.23	0.92
Recall that malaria diagnosis was made	0.82	0.86	0.66	1.00	0.82
Recall of any anti-malarial given^b^	0.64	0.65	0.60	0.63	0.69
Recall of ACT given^b^	0.53	0.51	0.51	0.62	0.47
Recall of ACT, corrected by visual aid	0.87	0.85	0.95	0.94	0.68
Recall of ACT, corrected by retained prescription	0.99	1.08	1.12	0.84	0.90
Recall of ACT, corrected by retained packaging	1.02	1.06	1.01	1.03	0.95
Recall of ACT, corrected by visual aid, prescription or retained packaging	1.20	1.26	1.28	1.17	1.09

A population-level bias is generally considered to be low if 0.75 < IF < 1.25; moderate if 0.5 < IF < 1.5; and high if IF < 0.5 or IF > 1.5, based on recommendations from the Child Health Epidemiology Reference Group (CHERG) and Improving Coverage Measurement (ICM) group

*ACT* artemisinin-based combination therapy, *CHW* community health worker, *IF* inflation factor

^a^Among those children tested

^b^Excludes treatment that caregiver reported receiving from a different facility or healthcare provider

## Discussion

This study investigated the validity of caregivers’ recall of the diagnosis and treatment received by children U5 years when seeking care for a fever (without severe disease), as measured by a household survey questionnaire in Mali. Recall of the child receiving a finger or heel prick, assumed to mean that the child was tested for malaria by microscopy or a rapid diagnostic test, had high sensitivity and specificity. However caregivers’ recall of treatment received by the child had low sensitivity, meaning that caregivers would falsely report during the interview that the child did not receive anti-malarials, when the gold standard measure indicates that they did receive an anti-malarial. Sensitivity was lower for receipt of ACT than for receipt of any type of anti-malarial, indicating that some caregivers knew their child was given an anti-malarial, but few correctly reported that the child received ACT. This finding was also clear from questionnaires, where some caregivers reported that their child received anti-malarial pills or an injection, but did not know the name of the drug.

The result of the finger or heel prick test (assumed to be a malaria test) and the health worker’s diagnosis for the child are not commonly included in DHS or MIS surveys. Our data suggest caregivers’ recall of a confirmed malaria diagnosis had high individual accuracy (based on AUC) and low population-level bias (based on an inflation factor) in this context, although specificity was only moderate due to caregivers incorrectly reporting the child had a positive malaria test result when the result was negative. These false positive responses may be related to incomplete adherence of health workers to negative malaria test results; instances of health workers prescribing anti-malarials to children regardless of a negative microscopy or RDT result were recorded during the study.

Indicators of individual- and population-level bias were worse for caregivers’ recall of a malaria diagnosis made by the health worker than for caregivers’ recall of a positive malaria test result, particularly among those caregivers who attended the public rural health facilities. It should be noted, however, that while only 49% of children recruited from public rural facilities were classified as cases (received ACT), 94% of children recruited at these facilities were diagnosed as malaria cases by health workers (presumptive and confirmed); this resulted in wide confidence intervals for estimates of specificity of caregivers’ recall of malaria diagnosis at public rural facilities.

Currently, standard household surveys do not measure the proportion of children receiving appropriate treatment for malaria because the denominator (children with confirmed malaria) has been considered too difficult to accurately measure in a survey setting. Alternative methods have been used to estimate appropriate treatment of malaria by restricting ACT coverage indicators to those children who had a positive *Plasmodium falciparum* histidine-rich protein 2 (HRP2) RDT result during the survey [5], since HRP2 antigen persists for several weeks after clearance of the infection [22, 23]. The results of this study suggest that caregivers’ recall of a positive malaria test result, in this context of high test positivity rates, may be sufficiently reliable to incorporate in standard population-based survey questionnaires. However, further validation of this indicator is recommended in settings with lower test positivity rates among febrile children, and in settings with low testing rates. Including an indicator of ACT coverage among children with confirmed malaria in standard population-based malaria surveys is increasingly feasible as countries continue to expand access to confirmatory diagnostics at public health facilities, at community-level, and in the private sector [4, 24].

Caregivers’ recall was substantially improved by correcting the initial response to the interview question asking what drugs the child received by including a visual aid, or requesting to see any retained prescription document or drug packaging retained from the facility or CHW visit. When based on the standard question response only, ACT recall had moderate individual-level accuracy and moderate population-level bias. However, by incorporating caregivers’ identification of ACT medicines from the photographs of common drugs prescribed for fever, or from prescriptions and packaging retained by the caregiver, AUC and IF estimates improved, indicating reduced individual- and population-level bias. Including all three additional methods to identify ACT medicines received by the child resulted in low to moderate population-level bias, and high individual-level accuracy. Inclusion of visual aids has been demonstrated to improve caregiver recall of pneumonia treatment in a survey in Pakistan and Bangladesh [25], and challenges in correctly identifying and differentiating anti-malarial drugs have previously been reported in Mali [15, 26]. Use of visual aids has been previously recommended to improve reporting validity of maternal and child health indicators in settings with multiple sources of medicines [6], and is included in DHS and MIS interviewer guidelines [27, 28], but has not been consistently included in all DHS and MIS surveys.

Improved ACT recall sensitivity (lower proportion of false negatives) was found to be associated with the treatment seeking event occurring with a CHW, having higher household wealth and being able to read. We hypothesize that improved recall among caregivers taking their child to a CHW could be a result of CHWs spending more time discussing the diagnosis and treatment with caregivers, or simply the smaller range of drugs available. Studies in Uganda reported communication gaps between patient and health workers, with limited explanation of diagnostic procedures and treatment provided [29], and higher perceived quality of care from CHWs than from public health facilities [30]. Literacy and education levels have been found to be associated with adherence to ACT [31], however, the previous recall validation study in Zambia found no association between sensitivity of ACT recall and caregivers’ education [7].

This study found some potential information bias in reporting of ACT received by the child during the period of SMC implementation, indicated by a significant decline in specificity of ACT recall during the SMC period compared with the pre-SMC period. During the SMC campaign in Mali, any child with fever is tested by RDT, and if the RDT is positive the child receives ACT instead of the SMC drug (sulfadoxine–pyrimethamine and amodiaquine). It is likely that some of these apparent false positive reports of ACT were the result of febrile and RDT positive children receiving ACT as a part of the SMC campaign, however it is not possible to confirm this without review of individual-level SMC campaign records. It should also be noted that not all countries implementing SMC have adopted this approach of testing febrile children with RDTs on the SMC campaign day.

This study specified that any child with signs of severe malaria or other severe disease should be excluded. However, signs and symptoms exhibited by participating children were not systematically recorded during the consultation or enrolment process. While both the study team members at health facilities and the clinicians conducting consultations at study locations were trained on the exclusion criteria, it is possible that some children with severe malaria were included in the study. Nevertheless, the rate of injectable anti-malarial use was high, and supports the conclusion that injectable anti-malarials were being provided to children with uncomplicated malaria.

Limitations of this study included the potential for caregivers’ recall to be biased as a result of contact with the study team during registration at the facility. While the specific aim of the study was not disclosed at the facility, interaction with the study team at the facility to enroll in a “child health study” could have biased recall. Similarly, the consultation record forms that provided the gold standard were completed by the health workers, without independent observation of the consultation to validate the information recorded. It was not possible to match child-level data from the study to SMC registers to confirm whether children received ACT during the SMC campaign, which may have biased ACT recall specificity downwards during the SMC period. Finally, this study includes a relatively small number of facilities, although from a variety of settings, so findings may not be directly applicable to different settings. However the results differ from those collected during a similar study in Zambia: a setting with high parasitological testing rates, high use of the public sector and no stock-outs of ACT medicines during the study period. While the Zambia study found that caregivers’ recall of ACT was good, our study indicates that in a high transmission setting where multiple anti-malarial drugs are commonly available and private sector use is higher, ACT recall has low sensitivity, unless additional tools such as visual aids are used. While the Zambia study directed the follow-up questionnaire to the child’s caregiver and asked separately who took the child to the health facility, the current study differed in that the questionnaire was directed to the individual who took the child for treatment. This could potentially have biased results from this study in Mali towards improved recall, compared to the study in Zambia.

This study found unexpectedly high use of non-ACT anti-malarials. The National Malaria Treatment Guidelines in Mali state that artemether–lumefantrine is the first line drug, with artesunate–amodiaquine as second-line. For severe malaria an injection of artesunate, artemether or quinine (depending on availability) is recommended, followed by a full course of ACT after the child is able to safely ingest the drugs. A stock-out of ACT was identified at the rural public health facilities during a supervision visit, which may have contributed to the use of injectable anti-malarials, however non-ACT anti-malarials were prescribed at all facility types. A subsequent publication will present detailed discussion of diagnosis and prescription practices recorded during this study.

## Conclusions

Indicators of parasitological testing for malaria and the result of that test were found to have high individual validity and low population bias in the examined sites in Mali, indicating that caregiver response to these questions in household surveys is comparable to the gold standard recorded by health workers. The slightly low specificity of positive test recall predicts that surveys would over-estimate this indicator in areas where a low proportion of tests are positive, however this finding should be confirmed in low transmission settings. Caregivers’ recall of ACT received by the child when assessed through an interview response only had low sensitivity, largely as a result of recall error (not knowing drug names) rather than information error (caregiver providing an incorrect response when they do not know). Sensitivity of ACT recall was substantially improved by including visual aids, review of prescriptions and any retained packaging. We recommend that future household surveys collecting standard malaria treatment indicators include at least one of these additional methods (visual aids, prescription or retained packaging review) to assist the caregiver in identifying if the child received ACT. These additional tools may be particularly beneficial in settings with low literacy among caregivers, and where a variety of anti-malarial medicines may be available from the public and/or private sector.

## Additional files

**Additional file 1.** Visual aid of drugs that may be prescribed for malaria in Mali.

**Additional file 2.** Participant enrolment over time bar chart.

**Additional file 3.** Sensitivity, specificity and accuracy of caregivers’ recall of diagnosis procedures and treatment received during consultation, by facility type.

**Additional file 4.** Random effects multivariate logistic regression models of sensitivity, specificity and accuracy of caregiver recall of treatment with ACT, corrected by visual aids, prescriptions and packaging.

**Additional file 5.** Summaries of sensitivity, specificity and accuracy of caregiver recall of treatment with ACT (assessed by questionnaire response only) by various facility, caregiver and child characteristics.

**Additional file 6.** Summaries of sensitivity, specificity and accuracy of caregiver recall of treatment with ACT, corrected by visual aid, prescription documents or retained packaging by various facility, caregiver and child characteristics.

**Additional file 7.** ROC curves for recall of blood test, recall of positive blood test, recall of malaria diagnosis by clinician, and recall of any anti-malarial being prescribed.

**Additional file 8.** ROC for corrected ACT recall by each adjustment question/method.
