# Supplementary material for: Measuring malaria diagnosis and treatment coverage in population-based surveys: a recall validation study in Mali among caregivers of febrile children under 5 years
Source: Malar J. 2019 Jan 3;18:3. doi: 10.1186/s12936-018-2636-3 (PMC6317217; doi:10.1186/s12936-018-2636-3)
Supplement: Supplementary file 1 — Additional file 1. Visual aid of drugs that may be prescribed for malaria in Mali. [file 12936_2018_2636_MOESM1_ESM.pdf]

Measuring malaria treatment coverage in population-based surveys: A recall validation study in Mali among caregivers of febrile children under five years

Ruth A. Ashton, Bakary Doumbia, Diadier Diallo, Thomas Druetz, Lia Florey, Cameron Taylor, Fred Arnold, Jules Mihigo, Diakalia Koné, Seydou Fomba, Erin Eckert, Thomas P. Eisele

Additional file 1 - Visual aid of drugs that may be prescribed to a child under five years with fever.

The visual aid was adapted from the version used in the Mali Malaria Indicator Survey in 2015. Prior to the survey, a range of antimalarial drugs were procured from private pharmacies in Bamako. This allowed inclusion of updated packaging designs in the visual aid, as well as inclusion of additional drug brands that were not previously included. A selection of tablets, suspensions and syrups were included, along with one example each of intravenous fluid and ampoule for injection. Each page of the visual aid was printed to fill one standard A4 sheet. Original images are available on request from the corresponding author.

A key to the drugs included is presented below, with detailed description of the drug and if it was included in the 2015 MIS. This key was not available to interviewers or to caregivers during the survey.

#	Drug name	Packaging description	Included in 2015 MIS visual aid?
A	Sulfadoxine/ Pyrimethamine + Amodiaquine	Drug used in Mali for SMC in 2017. New dispersible formulation for 12-59 months age. Packaging for <12 months age has is similar design but orange color.	No
B	Sulfadoxine/ Pyrimethamine	Maloxine brand. Pills in tear-to-open pouches, not blister packs.	Yes, but updated for new packaging
C	Sulfadoxine/ Pyrimethamine	Unbranded zip-lock bag of 3x white circular pills. Not clearly identifiable as SP	Yes
D	Quinine	Unbranded package of multiple small white circular pills. Quinine written on zip-lock bag	Yes
E	*Kunbileni*	Red & yellow capsules, active ingredient unknown. Commonly sold by ambulant vendors	Yes
F	*Kunbileni jeman*	White capsules, active ingredient unknown. Commonly sold by ambulant vendors	Yes
G	Artemether lumefantrine	Coartem brand. Image includes standard tablet packaging, and dispersible tablet packaging. 6 yellow pills in blister pack (infant dose).	Yes
H	Artesunate + amodiaquine	Generic brand, co-packaged blister of 12 yellow and 12 white pills	No

#	Drug name	Packaging description	Included in 2015 MIS visual aid?
J	Sulfadoxine/ Pyrimethamine + Artesunate	AsunateDenk box and blister pack, and Co-Arinate FDC box.	No
K	Dihydroartemisin in + piperaquine	Malacur box and blister pack. Co-formulated pills.	No
L	Aspirin	Generic aspirin in blister strip.	Yes
M	Paracetamol	Genetic paracetamol in blister strip.	Yes
N	Infusion set	Unidentified IV infusion	Yes
O	Quinine injectable	Unnamed ampoule. Name of drug not visible	Yes
P	Artemether lumefantrine suspensions	Co-artesiane and Artome brands. Boxes and bottles shown. Two brands of a wide selection of artemether lumefantrine suspensions available at Bamako pharmacies. The most distinctive packaging was chosen for images.	No
Q	Amodiaquine syrup	Camoquin brand syrup. Box and bottle shown.	No
R	Artemether lumefantrine	Coartem brand. Image shows dispersible tablets in easy-to-understand packaging. Infant (yellow) and toddler (blue) doses included.	Not this packaging
S	Chloroquine	Nirupquin brand. Drug name not clear on packaging. Blister strip of 10 white pills.	Yes
T	Artemether lumefantrine	Laritem brand. Alternative QA-ACT found in pharmacy in Bamako. Blister pack of 6 pills	No
U	Amodiaquine	Generic brand, clearly labelled as Amodiaquine. Blister pack of 12 pale yellow pills.	Yes

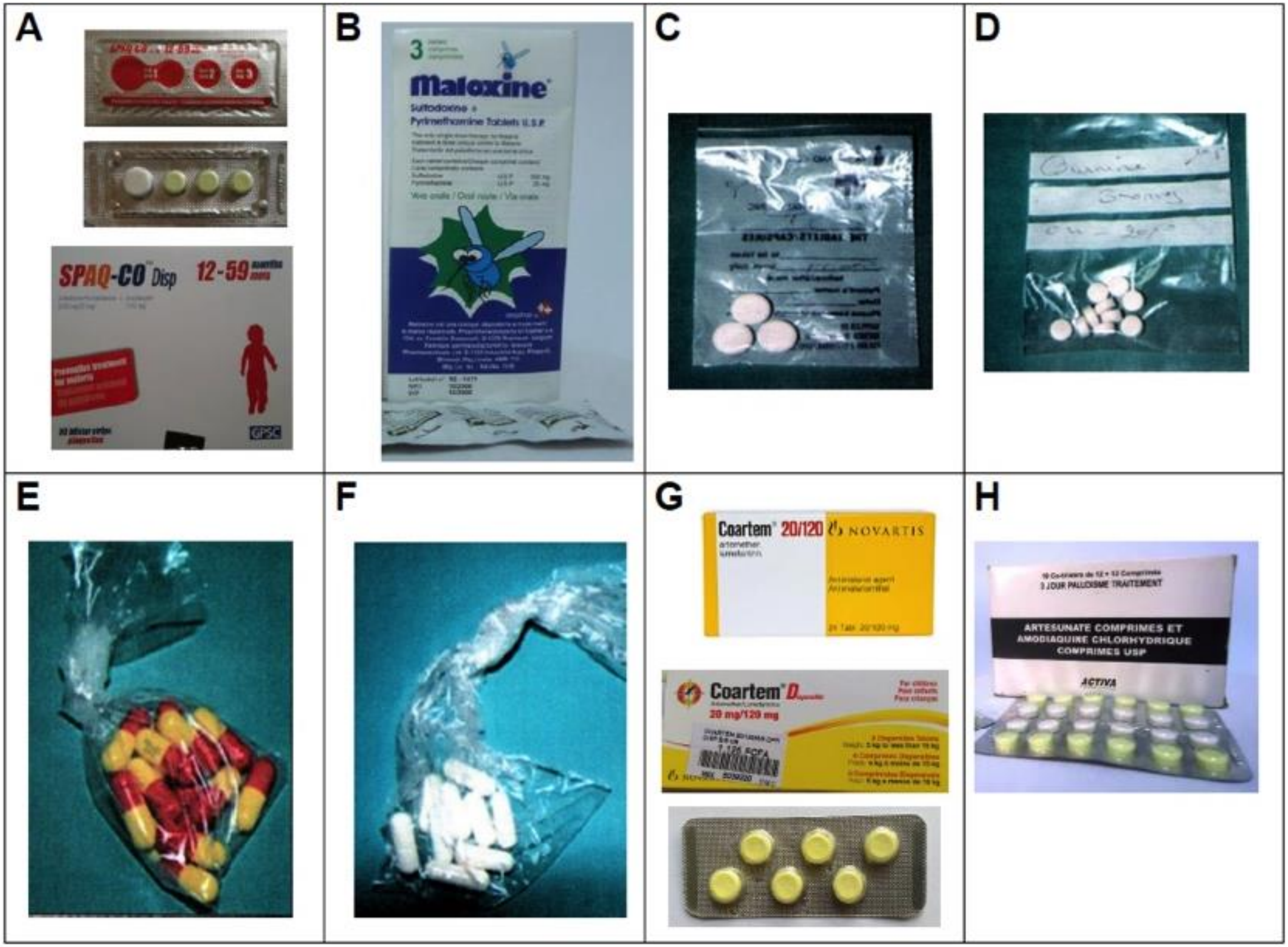
A
SPAQ-CO Disp
12-59
B
Maloxine
C
D
E
F
G
Coartem 20/120
NOVARTIS
Coartem D
20 mg/120 mg
H
ARTESUNATE COMPRIMES ET
AMODIAQUINE CHLORHYDRIQUE
COMPRIMES USP
ACTIVA

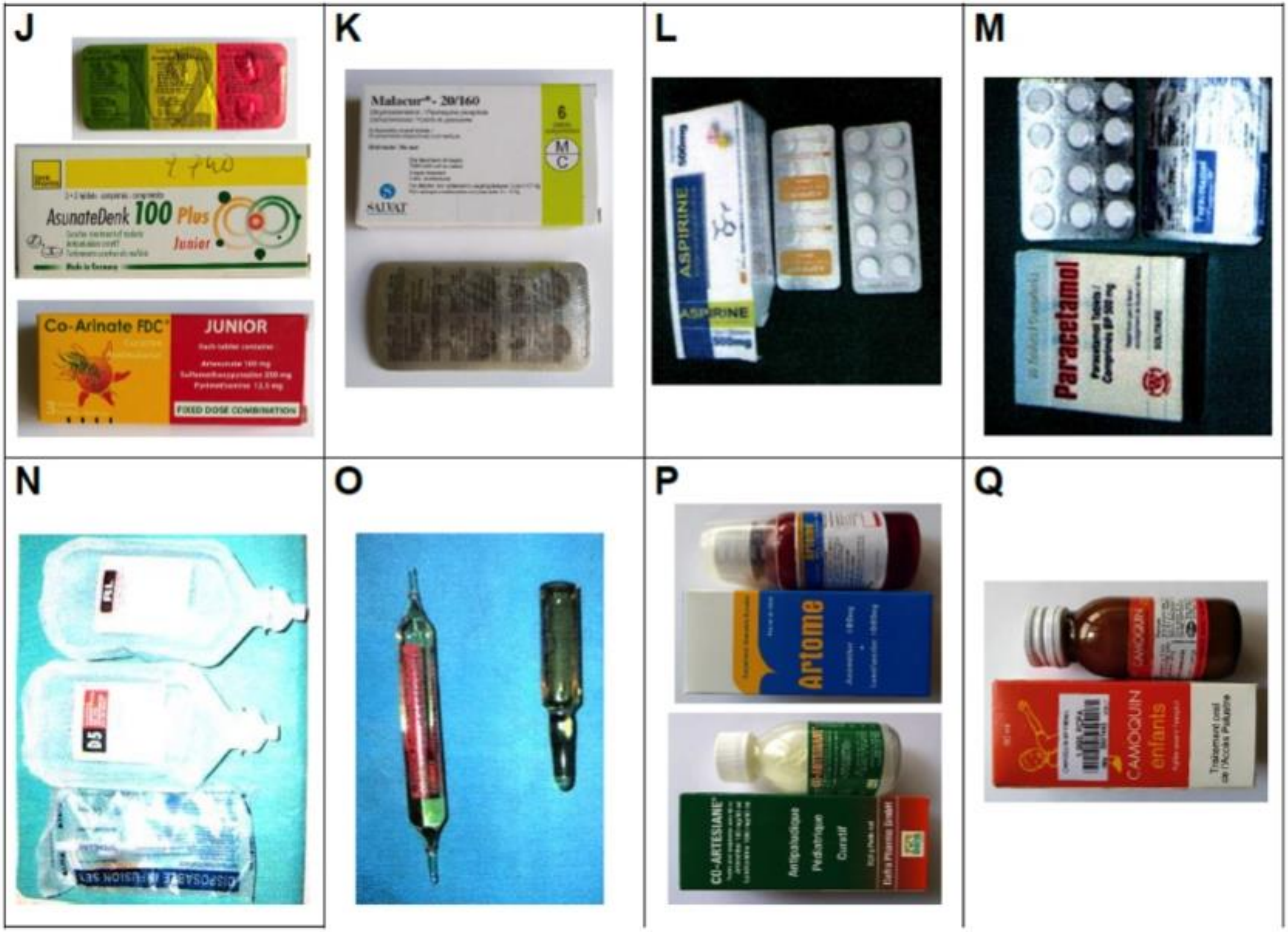
J
AsunateDenk 100 Plus
Junior
Co-Arinate FDC
JUNIOR
FIXED DOSE COMBINATION
K
Malacur® - 20/160
SALVAT
L
ASPIRINE
M
Paracetamol
N
D5
O
P
Artome
CO-ARTESIANE
Antipaludique
Pédiatrique
Curatif
Q
CAMOQUIN
enfants
Traitement oral de l'Accès Palustre

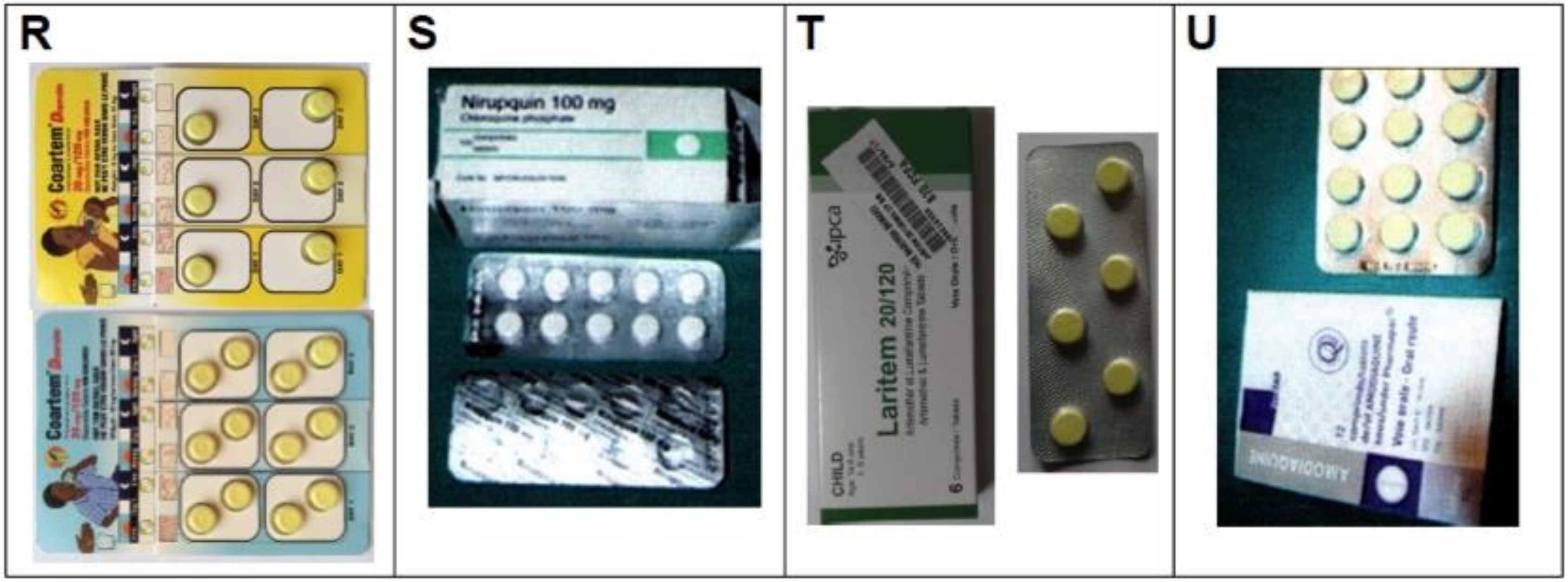
R
S
T
U
Coartem
Coartem
Nirupquin 100 mg
ipca
Laritem 20/120
CHILD
