# Supplementary material for: Measuring malaria diagnosis and treatment coverage in population-based surveys: a recall validation study in Mali among caregivers of febrile children under 5 years
Source: Malar J. 2019 Jan 3;18:3. doi: 10.1186/s12936-018-2636-3 (PMC6317217; doi:10.1186/s12936-018-2636-3)

Measuring malaria treatment coverage in population-based surveys: A recall validation study in Mali among caregivers of febrile children under five years

*Ruth A. Ashton, Bakary Doumbia, Diadier Diallo, Thomas Druetz, Lia Florey, Cameron Taylor, Fred Arnold, Jules Mihigo, Diakalia Koné, Seydou Fomba, Erin Eckert, Thomas P. Eisele*

Additional file 2

Stacked bar chart describing daily enrolment of cases (caregivers of children who received ACT at the health facility or CHW site) and controls (caregivers of children who did not receive ACT at the health facility or CHW site).

The 2017 SMC campaign in urban Sikasso began on 28 July, in Niena district SMC began on 31 July, and in urban Bamako SMC began on 10 August. All study participants from the pre-SMC period were recruited and completed their follow-up interview between 6 July and 27 July 2017. Recruitment stopped in August due to reduced accessibility during the peak rainy season. Participants were recruited for the during-SMC period starting 7 September, with all follow-up interviews completed by 5 November 2017.


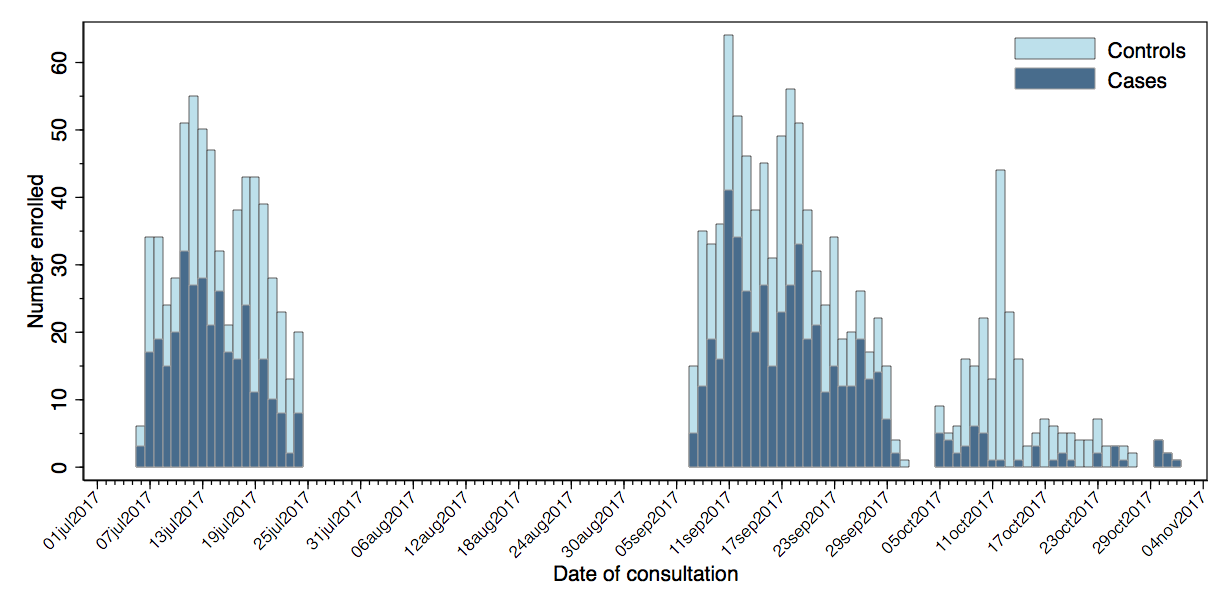

Supplement: Supplementary file 2 — Additional file 2. Participant enrolment over time bar chart. [file 12936_2018_2636_MOESM2_ESM.docx]
