# Supplementary material for: Measuring malaria diagnosis and treatment coverage in population-based surveys: a recall validation study in Mali among caregivers of febrile children under 5 years
Source: Malar J. 2019 Jan 3;18:3. doi: 10.1186/s12936-018-2636-3 (PMC6317217; doi:10.1186/s12936-018-2636-3)
Supplement: Supplementary file 3 — Additional file 3. Sensitivity, specificity and accuracy of caregivers’ recall of diagnosis procedures and treatment received during consultation, by facility type. [file 12936_2018_2636_MOESM3_ESM.docx]

Measuring malaria treatment coverage in population-based surveys: A recall validation study in Mali among caregivers of febrile children under five years

*Ruth A. Ashton, Bakary Doumbia, Diadier Diallo, Thomas Druetz, Lia Florey, Cameron Taylor, Fred Arnold, Jules Mihigo, Diakalia Koné, Seydou Fomba, Erin Eckert, Thomas P. Eisele*

Additional file 3

Sensitivity, specificity and accuracy of caregivers’ recall of diagnosis procedures and treatment received during consultation, by facility type.

|  | Sensitivity^1^ | | | Specificity^2^ | | | Accuracy^3^ | | |
| --- | --- | --- | --- | --- | --- | --- | --- | --- | --- |
|  | N | % | 95% CI | N | % | 95% CI | N | % | 95% CI |
| **Public urban facilities** |  |  |  |  |  |  |  |  |  |
| Recall of fever in past 2 weeks^4^ | 421 | 100.0 | 100.0,100.0 | 0 | - | - | 421 | 100.0 | 100.0,100.0 |
| Recall of finger/heel stick | 352 | 96.6 | 94.7,98.5 | 69 | 75.4 | 64.9,85.8 | 421 | 93.1 | 90.7,95.5 |
| Recall of positive malaria test result^5^ | 219 | 95.0 | 92.1,97.9 | 72 | 51.4 | 39.6,63.2 | 291 | 84.2 | 80.0,88.4 |
| Recall that malaria diagnosis was made | 347 | 79.3 | 75.0,83.5 | 73 | 71.2 | 60.6,81.9 | 420 | 77.9 | 73.9,81.8 |
| Recall of any antimalarial given^6^ | 371 | 61.7 | 56.8,66.7 | 47 | 74.5 | 61.5,87.4 | 418 | 63.2 | 58.5,67.8 |
| Recall of ACT given^6^ | 225 | 41.8 | 35.3,48.3 | 186 | 88.7 | 84.1,93.3 | 411 | 63.0 | 58.3,67.7 |
| Corrected recall of ACT given^7^ | 231 | 90.9 | 87.2,94.6 | 190 | 57.4 | 50.3,64.5 | 421 | 75.8 | 71.7,79.9 |
|  |  |  |  |  |  |  |  |  |  |
| **Public rural facilities** |  |  |  |  |  |  |  |  |  |
| Recall of fever in past 2 weeks^4^ | 420 | 99.3 | 98.5,100.0 | 0 | - | - | 420 | 99.3 | 98.5,100.0 |
| Recall of finger/heel stick | 113 | 73.5 | 65.2,81.7 | 296 | 86.1 | 82.2,90.1 | 409 | 82.6 | 79.0,86.3 |
| Recall of positive malaria test result^5^ | 34 | 97.1 | 91.1,100.0 | 2 | 50.0 | - | 36 | 94.4 | 86.6,100.0 |
| Recall that malaria diagnosis was made | 392 | 63.8 | 59.0,68.6 | 23 | 56.5 | 34.6,78.4 | 415 | 63.4 | 58.7,68.0 |
| Recall of any antimalarial given^6^ | 401 | 58.9 | 54.0,63.7 | 16 | 62.5 | 35.9,89.1 | 417 | 59.0 | 54.3,63.7 |
| Recall of ACT given^6^ | 200 | 39.0 | 32.2,45.8 | 213 | 88.3 | 83.9,92.6 | 413 | 64.4 | 59.8,69.0 |
| Corrected recall of ACT given^7^ | 204 | 94.6 | 91.5,97.7 | 213 | 68.5 | 62.3,74.8 | 417 | 81.3 | 77.5,85.1 |
|  |  |  |  |  |  |  |  |  |  |
| **CHWs** |  |  |  |  |  |  |  |  |  |
| Recall of fever in past 2 weeks^4^ | 425 | 99.8 | 99.3,100.0 | 0 | - | - | 425 | 99.8 | 99.3,100.0 |
| Recall of finger/heel stick | 281 | 91.5 | 88.2,94.7 | 142 | 72.5 | 65.1,80.0 | 423 | 85.1 | 81.7,88.5 |
| Recall of positive malaria test result^5^ | 140 | 97.9 | 95.4,100.0 | 68 | 64.7 | 53.1,76.4 | 208 | 87.0 | 82.4,91.6 |
| Recall that malaria diagnosis was made | 286 | 83.6 | 79.2,87.9 | 132 | 65.2 | 56.9,73.4 | 418 | 77.8 | 73.7,81.8 |
| Recall of any antimalarial given^6^ | 279 | 56.3 | 50.4,62.1 | 128 | 83.6 | 77.1,90.1 | 407 | 64.9 | 60.2,69.5 |
| Recall of ACT given^6^ | 195 | 55.9 | 48.9,62.9 | 205 | 93.2 | 89.7,96.7 | 400 | 75.0 | 70.7,79.3 |
| Corrected recall of ACT given^7^ | 212 | 97.2 | 94.9,99.4 | 209 | 81.3 | 76.0,86.7 | 421 | 89.3 | 86.3,92.3 |
|  |  |  |  |  |  |  |  |  |  |
| **Private urban facilities** |  |  |  |  |  |  |  |  |  |
| Recall of fever in past 2 weeks^4^ | 336 | 99.7 | 99.1,100.0 | 0 | - | - | 336 | 99.7 | 99.1,100.0 |
| Recall of finger/heel stick | 46 | 97.8 | 93.4,100.0 | 289 | 94.1 | 91.4,96.8 | 335 | 94.6 | 92.2,97.1 |
| Recall of positive malaria test result^5^ | 28 | 96.4 | 89.1,100.0 | 12 | 83.3 | 58.6,100.0 | 40 | 92.5 | 84.0,100.0 |
| Recall that malaria diagnosis was made | 188 | 72.9 | 66.5,79.3 | 147 | 88.4 | 83.2,93.7 | 335 | 79.7 | 75.4,84.0 |
| Recall of any antimalarial given^6^ | 184 | 58.2 | 51.0,65.3 | 145 | 86.9 | 81.3,92.5 | 329 | 70.8 | 65.9,75.8 |
| Recall of ACT given^6^ | 135 | 33.3 | 25.3,41.4 | 190 | 90.5 | 86.3,94.7 | 325 | 66.8 | 61.6,71.9 |
| Corrected recall of ACT given^7^ | 140 | 79.3 | 72.5,86.1 | 195 | 79.0 | 73.2,84.7 | 335 | 79.1 | 74.7,83.5 |

^1^Sensitivity calculated as total true positives (not shown) divided by the number of true positives and false negatives (N); ^2^Specificity calculated as the total true negatives (not shown) divided by the number of true negatives and false positives (N); ^3^Accuracy calculated as the sum of true positives and true negatives (not shown) divided by sum of true positives, true negatives, false positives and true negatives (N); ^4^Fever was an inclusion criteria, therefore the gold standard for fever was inclusion in the study; ^5^Among those children tested; ^6^Excludes treatment that caregiver reported receiving from a different facility or healthcare provider; ^7^Recall of ACT from interview corrected to include instances where caregiver identified ACT from the visual aid, or had retained a prescription including ACT or ACT packaging from the child’s consultation.
