# Supplementary material for: Measuring malaria diagnosis and treatment coverage in population-based surveys: a recall validation study in Mali among caregivers of febrile children under 5 years
Source: Malar J. 2019 Jan 3;18:3. doi: 10.1186/s12936-018-2636-3 (PMC6317217; doi:10.1186/s12936-018-2636-3)
Supplement: Supplementary file 4 — Additional file 4. Random effects multivariate logistic regression models of sensitivity, specificity and accuracy of caregiver recall of treatment with ACT, corrected by visual aids, prescriptions and packaging. [file 12936_2018_2636_MOESM4_ESM.docx]

Measuring malaria treatment coverage in population-based surveys: A recall validation study in Mali among caregivers of febrile children under five years

*Ruth A. Ashton, Bakary Doumbia, Diadier Diallo, Thomas Druetz, Lia Florey, Cameron Taylor, Fred Arnold, Jules Mihigo, Diakalia Koné, Seydou Fomba, Erin Eckert, Thomas P. Eisele*

**Additional file 4**

Random effects multivariate logistic regression models of sensitivity, specificity and accuracy of caregiver recall of treatment with ACT, corrected by visual aids, prescriptions and packaging: associations with follow-up and socio-demographic characteristics

|  | Sensitivity | | Specificity | | Accuracy | |
| --- | --- | --- | --- | --- | --- | --- |
|  | AOR (95% CI) | p-value | AOR (95% CI) | p-value | AOR (95% CI) | p-value |
| Type of facility |  |  |  |  |  |  |
| Public urban | 1.00 |  | 1.00 |  | 1.00 |  |
| Public rural | 2.32 (0.77,7.03) | 0.137 | 2.59 (0.60,11.18) | 0.201 | 1.43 (0.85,2.40) | 0.174 |
| CHW | 3.13 (0.88,11.14) | 0.077 | 8.65 (2.28,32.89) | 0.002 | 3.06 (1.73,5.42) | <0.001 |
| Private urban | 0.95 (0.39,2.27) | 0.903 | 1.60 (0.35,7.31) | 0.543 | 1.49 (0.92,2.41) | 0.105 |
| Region |  |  |  |  |  |  |
| Bamako | 0.29 (0.12,0.72) | 0.008 | 4.20 (0.86,20.59) | 0.077 | 0.89 (0.56,1.40) | 0.607 |
| Sikasso | 1.00 |  | 1.00 |  | 1.00 |  |
| Time period |  |  |  |  |  |  |
| Before SMC | 1.00 |  | 1.00 |  | 1.00 |  |
| During SMC | 0.90 (0.47,1.72) | 0.754 | 0.53 (0.35,0.81) | 0.003 | 0.67 (0.49,0.91) | 0.010 |
| Child’s age in years |  |  |  |  |  |  |
| <1 | 0.38 (0.12,1.20) | 0.099 | 2.61 (1.44,7.73) | 0.001 | 1.35 (0.84,2.19) | 0.215 |
| 1 | 0.37 (0.14,1.03) | 0.058 | 1.36 (0.75,2.46) | 0.314 | 0.98 (0.63,1.53) | 0.922 |
| 2 | 0.50 (0.18,1.37) | 0.175 | 1.42 (0.81,2.51) | 0.225 | 1.09 (0.70,1.68) | 0.702 |
| 3 | 0.44 (0.16,1.22) | 0.113 | 1.96 (1.07,3.59) | 0.029 | 1.21 (0.76,1.91) | 0.418 |
| 4 | 1.00 |  | 1.00 |  | 1.00 |  |
| Child’s sex |  |  |  |  |  |  |
| Male | 1.17 (0.64,2.13) | 0.611 | 0.85 (0.58,1.23) | 0.387 | 0.86 (0.65,1.14) | 0.288 |
| Female | 1.00 |  | 1.00 |  | 1.00 |  |
| Days to follow-up |  |  |  |  |  |  |
| 1-7 | 1.00 |  | 1.00 |  | 1.00 |  |
| 8-14 | 1.29 (0.69,2.44) | 0.426 | 0.88 (0.61,1.28) | 0.510 | 1.00 (0.75,1.33) | 0.991 |
| Caregiver’s sex |  |  |  |  |  |  |
| Male | 0.36 (0.17,0.79) | 0.010 | 1.59 (0.91,2.77) | 0.104 | 1.01 (0.67,1.52) | 0.975 |
| Female | 1.00 |  | 1.00 |  | 1.00 |  |
| Caregiver’s age |  |  |  |  |  |  |
| 18-24 | 0.91 (0.27,3.13) | 0.882 | 1.17 (0.48,2.85) | 0.723 | 0.83 (0.43,1.60) | 0.577 |
| 25-34 | 1.14 (0.37,3.54) | 0.822 | 0.84 (0.36,1.95) | 0.690 | 0.80 (0.43,1.48) | 0.479 |
| 35-44 | 0.86 (0.26,2.87) | 0.812 | 0.97 (0.40,2.35) | 0.949 | 0.88 (0.46,1.68) | 0.698 |
| ≥45 | 1.00 |  | 1.00 |  | 1.00 |  |
| Socio-economic status |  |  |  |  |  |  |
| 1 - Poorest | 1.08 (0.28,4.20) | 0.907 | 1.86 (0.75,4.65) | 0.183 | 1.45 (0.75,2.79) | 0.272 |
| 2 | 1.31 (0.36,4.76) | 0.680 | 2.32 (0.97,5.54) | 0.058 | 1.58 (0.84,2.95) | 0.152 |
| 3 | 2.12 (0.73,6.14) | 0.166 | 1.84 (0.86,3.95) | 0.116 | 1.66 (0.96,2.86) | 0.071 |
| 4 | 1.97 (0.84,4.62) | 0.119 | 1.10 (0.58,2.08) | 0.767 | 1.48 (0.94,2.33) | 0.091 |
| 5 - Wealthiest | 1.00 |  | 1.00 |  | 1.00 |  |
| Literacy of caregiver |  |  |  |  |  |  |
| Cannot read at all | 0.72 (0.32,1.67) | 0.448 | 0.63 (0.37,1.08) | 0.093 | 0.64 (0.43,0.96) | 0.029 |
| Can read a little | 0.29 (0.12,0.71) | 0.006 | 0.85 (0.41,1.73) | 0.649 | 0.66 (0.40,1.10) | 0.112 |
| Can read all of sentence | 1.00 |  | 1.00 |  | 1.00 |  |

AOR = Adjusted odds ratios; CHW = community health worker; SMC = seasonal malaria chemoprevention
