# Supplementary material for: Measuring malaria diagnosis and treatment coverage in population-based surveys: a recall validation study in Mali among caregivers of febrile children under 5 years
Source: Malar J. 2019 Jan 3;18:3. doi: 10.1186/s12936-018-2636-3 (PMC6317217; doi:10.1186/s12936-018-2636-3)
Supplement: Supplementary file 6 — Additional file 6. Summaries of sensitivity, specificity and accuracy of caregiver recall of treatment with ACT, corrected by visual aid, prescription documents or retained packaging by various facility, caregiver and child characteristics. [file 12936_2018_2636_MOESM6_ESM.docx]

Measuring malaria treatment coverage in population-based surveys: A recall validation study in Mali among caregivers of febrile children under five years

*Ruth A. Ashton, Bakary Doumbia, Diadier Diallo, Thomas Druetz, Lia Florey, Cameron Taylor, Fred Arnold, Jules Mihigo, Diakalia Koné, Seydou Fomba, Erin Eckert, Thomas P. Eisele*

**Additional file 6**

Summaries of sensitivity, specificity and accuracy of caregiver recall of treatment with ACT, corrected by visual aid, prescription documents or retained packaging by various facility, caregiver and child characteristics. Chi-squared test p-value is presented, or Fisher’s exact p-value where cell counts are less than 10, for differences in sensitivity, specificity or accuracy by the facility, caregiver and child characteristics. Drugs reportedly received from sources other than the recruiting health facility or CHW are excluded.

|  | Sensitivity | | Specificity | | Accuracy | | Sample size |
| --- | --- | --- | --- | --- | --- | --- | --- |
|  | % | 95% CI | % | 95% CI | % | 95% CI |  |
| Type of facility |  |  |  |  |  |  |  |
| Public urban | 90.9 | 87.2,94.6 | 57.4 | 50.3,64.5 | 75.8 | 71.7,79.9 | 421 |
| Public rural | 94.6 | 91.5,97.7 | 68.5 | 62.3,74.8 | 81.3 | 77.5,85.1 | 417 |
| CHW | 97.2 | 94.9,99.4 | 81.3 | 76.0,86.7 | 89.3 | 86.3,92.3 | 421 |
| Private urban | 79.3 | 72.5,86.1 | 79.0 | 73.2,84.7 | 79.1 | 74.7,83.5 | 335 |
|  |  | p<0.001^†^ |  | p<0.001 |  | p<0.001 |  |
| Region |  |  |  |  |  |  |  |
| Bamako | 76.5 | 69.7,83.3 | 76.3 | 71.5,81.1 | 76.4 | 72.5,80.3 | 457 |
| Sikasso | 95.1 | 93.4,96.8 | 69.0 | 64.9,73.0 | 83.6 | 81.4,85.7 | 1137 |
|  |  | p<0.001 |  | p=0.025 |  | p=0.001 |  |
| Facility environment |  |  |  |  |  |  |  |
| Rural | 86.5 | 83.0,90.0 | 68.3 | 63.6,73.0 | 77.2 | 74.3,80.2 | 756 |
| Urban | 95.9 | 94.0,97.8 | 74.9 | 70.7,79.0 | 85.3 | 82.9,87.7 | 838 |
|  |  | p<0.001 |  | p=0.038 |  | p<0.001 |  |
| Time period |  |  |  |  |  |  |  |
| Before SMC | 94.0 | 91.3,96.6 | 72.7 | 67.5,77.8 | 83.8 | 80.8,86.7 | 604 |
| During SMC | 89.8 | 87.1,92.6 | 71.2 | 67.3,75.1 | 80.1 | 77.6,82.6 | 990 |
|  |  | p=0.046 |  | p=0.666 |  | p=0.067 |  |
| Child’s age in years |  |  |  |  |  |  |  |
| <1 | 90.7 | 85.2,96.3 | 81.4 | 75.9,87.0 | 84.8 | 80.7,88.8 | 302 |
| 1 | 89.1 | 84.5,93.8 | 66.4 | 58.9,74.0 | 78.6 | 74.1,83.1 | 327 |
| 2 | 90.3 | 86.0,94.6 | 70.7 | 64.0,77.4 | 80.6 | 76.5,84.7 | 366 |
| 3 | 91.2 | 86.5,95.8 | 72.1 | 64.6,79.7 | 81.9 | 77.4,86.4 | 287 |
| 4 | 95.9 | 92.9,98.9 | 65.0 | 57.0,73.0 | 82.1 | 77.8,86.3 | 312 |
|  |  | p=0.146^†^ |  | p=0.006 |  | p=0.372 |  |
| Child’s sex |  |  |  |  |  |  |  |
| Male | 92.3 | 89.7,94.9 | 70.4 | 66.2,74.6 | 80.8 | 78.2,83.4 | 870 |
| Female | 90.6 | 87.6,93.6 | 73.5 | 68.9,78.1 | 82.3 | 79.5,85.1 | 724 |
|  |  | p=0.406 |  | p=0.331 |  | p=0.438 |  |
| Days to follow-up |  |  |  |  |  |  |  |
| 1-7 | 91.8 | 89.3,94.2 | 73.2 | 69.4,77.1 | 82.3 | 79.9,84.7 | 993 |
| 8-14 | 91.1 | 87.8,94.3 | 69.2 | 64.0,74.5 | 80.2 | 77.0,83.4 | 601 |
|  |  | p=0.735 |  | p=0.223 |  | p=0.301 |  |
| Caregiver’s sex |  |  |  |  |  |  |  |
| Male | 88.5 | 83.5,93.6 | 73.5 | 65.9,81.1 | 81.7 | 77.2,86.1 | 289 |
| Female | 92.2 | 90.1,94.3 | 71.4 | 68.0,74.8 | 81.5 | 79.3,83.6 | 1305 |
|  |  | p=0.139 |  | p=0.628 |  | p=0.935 |  |
| Caregiver’s age |  |  |  |  |  |  |  |
| 18-24 | 90.8 | 86.7,94.9 | 73.3 | 67.3,79.2 | 81.6 | 77.8,85.4 | 413 |
| 25-34 | 91.8 | 89.0,94.6 | 70.5 | 65.9,75.2 | 81.1 | 78.2,83.9 | 739 |
| 35-44 | 91.9 | 87.7,96.2 | 70.4 | 63.5,77.4 | 80.9 | 76.6,85.2 | 330 |
| ≥45 | 90.6 | 83.3,98.0 | 79.2 | 67.2,91.1 | 85.7 | 79.1,92.3 | 112 |
|  |  | p=0.952^†^ |  | p=0.576 |  | p=0.683 |  |
| Socio-economic status |  |  |  |  |  |  |  |
| 1 - Poorest | 94.4 | 90.9,98.0 | 73.8 | 66.7,81.0 | 84.6 | 80.5,88.6 | 311 |
| 2 | 95.2 | 91.6,98.7 | 76.2 | 69.7,82.7 | 85.0 | 81.0,89.0 | 313 |
| 3 | 93.9 | 90.2,97.6 | 71.5 | 64.2,78.8 | 83.2 | 79.0,87.3 | 315 |
| 4 | 89.1 | 84.4,93.8 | 67.1 | 59.3,75.0 | 79.3 | 74.8,83.8 | 314 |
| 5 - Wealthiest | 85.0 | 78.8,91.3 | 69.1 | 62.5,75.8 | 75.6 | 70.8,80.3 | 315 |
|  |  | p=0.011^†^ |  | p=0.402 |  | p=0.009 |  |
| Caregiver education level |  |  |  |  |  |  |  |
| None | 94.7 | 92.4,97.0 | 71.1 | 66.6,75.7 | 82.4 | 79.7,85.2 | 746 |
| Primary | 89.2 | 84.8,93.6 | 76.8 | 70.6,82.9 | 83.2 | 79.4,86.9 | 380 |
| Secondary or higher | 88.5 | 84.3,92.6 | 69.0 | 63.0,75.0 | 78.8 | 75.0,82.5 | 466 |
|  |  | p=0.013 |  | p=0.197 |  | p=0.177 |  |
| Literacy of caregiver |  |  |  |  |  |  |  |
| Cannot read at all | 94.6 | 92.4,96.8 | 71.2 | 66.9,75.6 | 82.8 | 80.2,85.4 | 826 |
| Can read a little | 82.2 | 73.2,91.2 | 75.3 | 65.5,85.2 | 78.7 | 72.0,85.3 | 150 |
| Can read all of sentence | 89.9 | 86.0,93.9 | 70.6 | 64.3,76.9 | 80.8 | 77.1,84.5 | 432 |
|  |  | p=0.001 |  | p=0.722 |  | p=0.398 |  |

†Fishers exact test used due to any cell count below 10
