# Supplementary material for: Measuring malaria diagnosis and treatment coverage in population-based surveys: a recall validation study in Mali among caregivers of febrile children under 5 years
Source: Malar J. 2019 Jan 3;18:3. doi: 10.1186/s12936-018-2636-3 (PMC6317217; doi:10.1186/s12936-018-2636-3)

Measuring malaria treatment coverage in population-based surveys: A recall validation study in Mali among caregivers of febrile children under five years

*Ruth A. Ashton, Bakary Doumbia, Diadier Diallo, Thomas Druetz, Lia Florey, Cameron Taylor, Fred Arnold, Jules Mihigo, Diakalia Koné, Seydou Fomba, Erin Eckert, Thomas P. Eisele*

**Additional file 7**

ROC curves for recall of blood test, recall of positive blood test, recall of malaria diagnosis by clinician, and recall of any antimalarial being prescribed


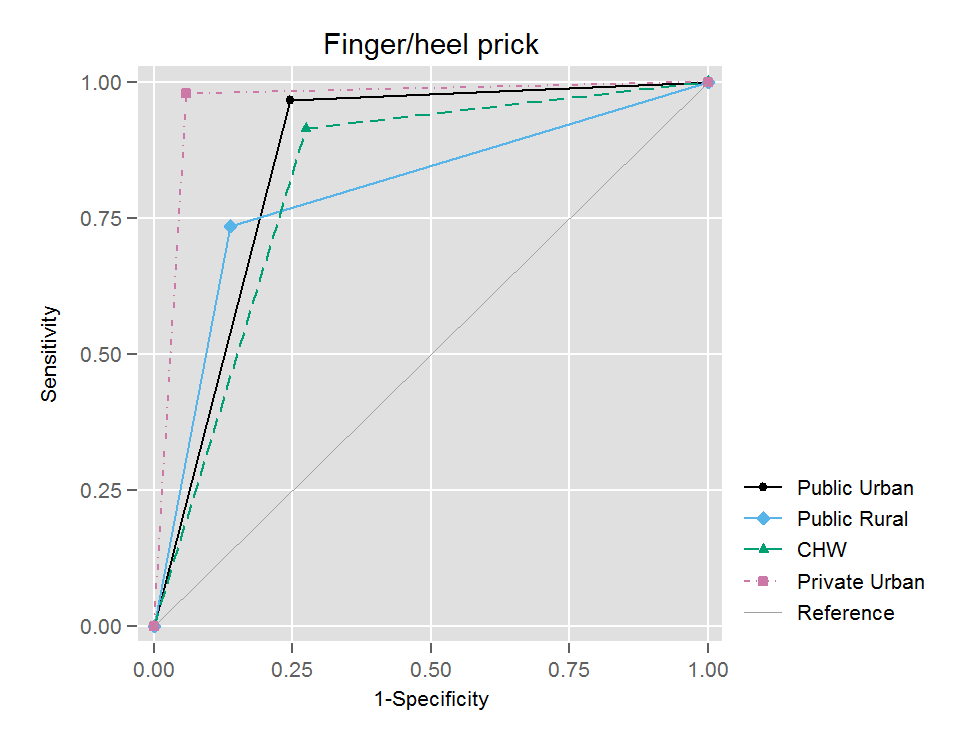


.
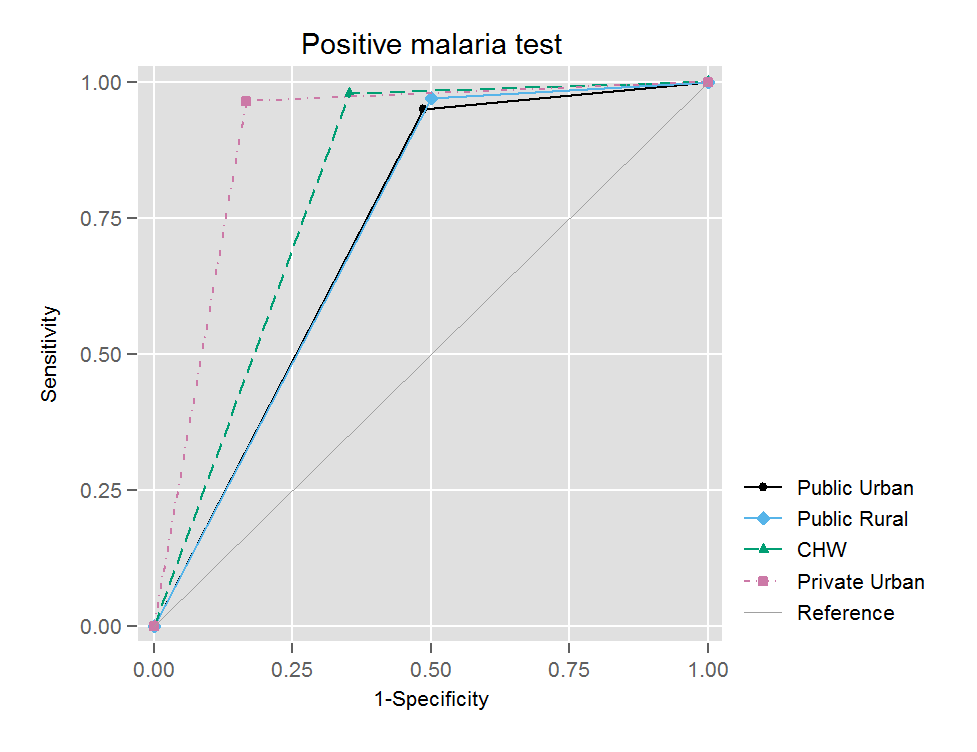


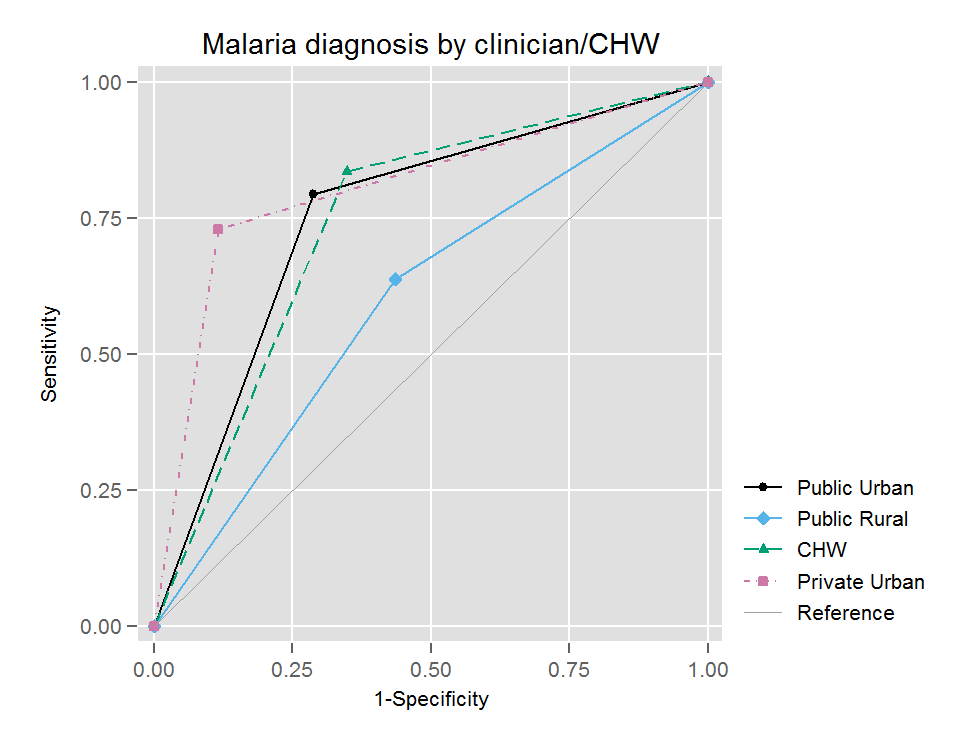


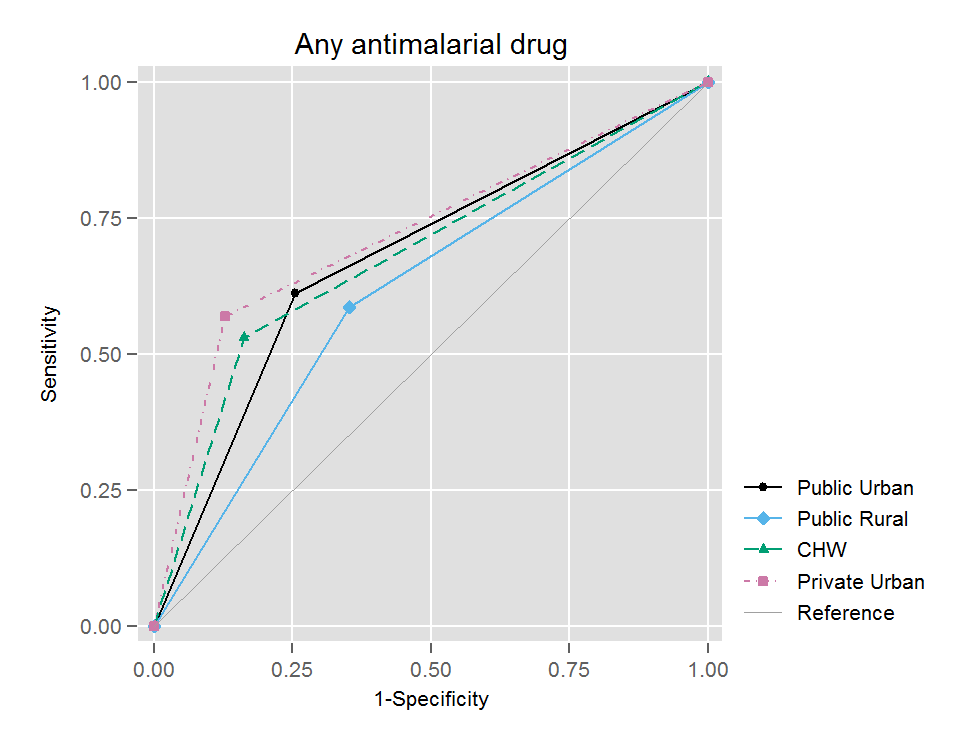

Supplement: Supplementary file 7 — Additional file 7. ROC curves for recall of blood test, recall of positive blood test, recall of malaria diagnosis by clinician, and recall of any anti-malarial being prescribed. [file 12936_2018_2636_MOESM7_ESM.docx]
