# Supplementary material for: Measuring malaria diagnosis and treatment coverage in population-based surveys: a recall validation study in Mali among caregivers of febrile children under 5 years
Source: Malar J. 2019 Jan 3;18:3. doi: 10.1186/s12936-018-2636-3 (PMC6317217; doi:10.1186/s12936-018-2636-3)

Measuring malaria treatment coverage in population-based surveys: A recall validation study in Mali among caregivers of febrile children under five years

*Ruth A. Ashton, Bakary Doumbia, Diadier Diallo, Thomas Druetz, Lia Florey, Cameron Taylor, Fred Arnold, Jules Mihigo, Diakalia Koné, Seydou Fomba, Erin Eckert, Thomas P. Eisele*

**Additional file 8**

ROC for adjusted ACT recall by each adjustment method


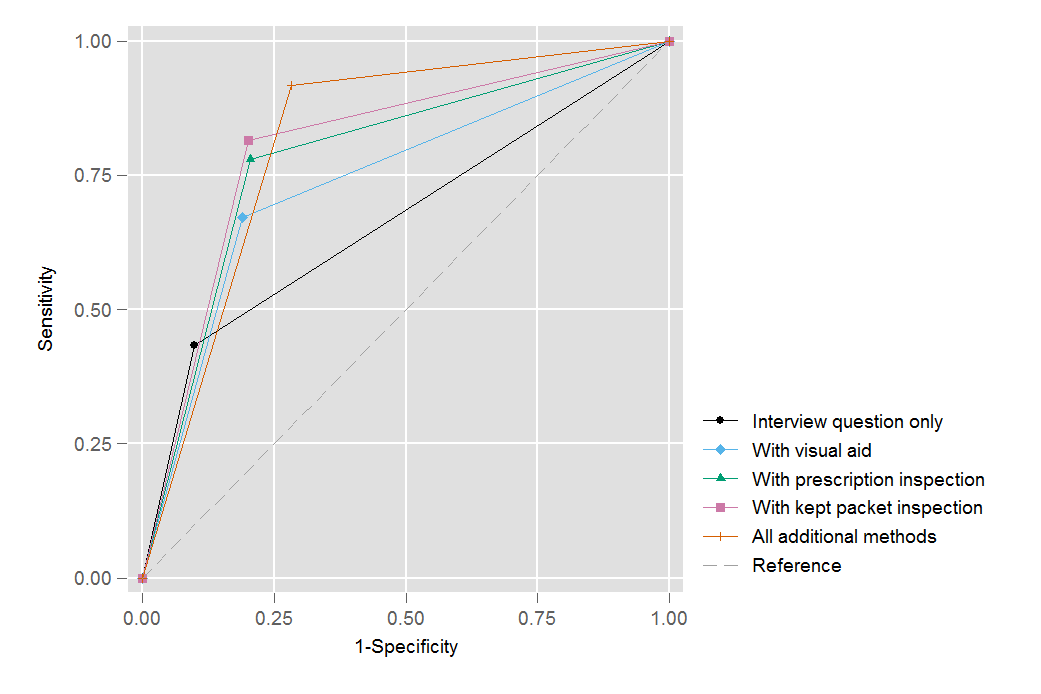

Supplement: Supplementary file 8 — Additional file 8. ROC for corrected ACT recall by each adjustment question/method. [file 12936_2018_2636_MOESM8_ESM.docx]
